# Delivery of polymeric nanostars for molecular imaging and endoradiotherapy through the enhanced permeability and retention (EPR) effect

**DOI:** 10.7150/thno.36777

**Published:** 2020-01-01

**Authors:** Jeroen A.C.M. Goos, Andrew Cho, Lukas M. Carter, Thomas R. Dilling, Maria Davydova, Komal Mandleywala, Simon Puttick, Abhishek Gupta, William S. Price, John F. Quinn, Michael R. Whittaker, Jason S. Lewis, Thomas P. Davis

**Affiliations:** 1 Department of Radiology, Memorial Sloan-Kettering Cancer Center, New York, USA.; 2 ARC Centre of Excellence in Convergent Bio-Nano Science & Technology, Monash Institute of Pharmaceutical Sciences, Monash University, Parkville, Australia.; 3 Department of Biochemistry & Structural Biology, Weill Cornell Graduate School, New York, USA.; 4 Weill Cornell/Rockefeller/Sloan Kettering Tri-Institutional MD-PhD Program, New York, USA.; 5 Probing Biosystems Future Science Platform, Commonwealth Scientific and Industrial Research Organisation, Herston, Australia.; 6 Nanoscale Organisation and Dynamics Group, Western Sydney University, Penrith, Australia.; 7 Department of Radiology, the Molecular Pharmacology Program and the Radiochemistry and Molecular Imaging Probes Core, Memorial Sloan Kettering Cancer Center, New York, USA.; 8 Departments of Radiology and Pharmacology, Weill Cornell Medical College, New York, USA.; 9 Australian Institute for Bioengineering and Nanotechnology, University of Queensland, St Lucia, Australia.

**Keywords:** star polymer, nanoparticle, EPR effect, imaging, therapy

## Abstract

Expression levels of biomarkers are generally unknown at initial diagnosis. The development of theranostic probes that do not rely on biomarker availability would expand therapy options for cancer patients, improve patient selection for nanomedicine and facilitate treatment of inoperable patients or patients with acquired therapy resistance. Herein, we report the development of star polymers, also known as nanostars, that allow for molecular imaging and/or endoradiotherapy based on passive targeting *via* the enhanced permeability and retention (EPR) effect.

**Methods:** We synthesised a star copolymer, consisting of 7-8 centre-cross-linked arms that were modified with Gd^3+^ for magnetic resonance imaging (MRI), and functionalised either with ^89^Zr for *in vivo* quantification and positron emission tomography (PET) imaging, or with ^177^Lu for endoradiotherapy. ^1^H longitudinal relaxivities were determined over a continuum of magnetic field strengths ranging from 0.24 mT - 0.94 T at 37 °C (nuclear magnetic relaxation dispersion (NMRD) profile) and *T*_1_-weighted MRI contrast enhancement was visualized at 3 T and 7 T. PET imaging and *ex vivo* biodistribution studies were performed in mice bearing tumours with high EPR (CT26) or low EPR (BxPC3) characteristics. Therapy studies were performed in mice with high EPR tumours and mean absorbed organ doses were estimated for a standard human model.

**Results:** The star copolymer with Gd^3+^ displayed a significantly superior contrast enhancement ability (*T*_1_ = 0.60 s) compared to the standard clinical contrast agent Gadovist (*T*_1_ = 1.0 s). Quantification of tumour accumulation using the radiolabelled nanostars in tumour-bearing mice demonstrated an exceptionally high uptake in tumours with high EPR characteristics (14.8 - 21.7 %ID/g). Uptake of the star polymers in tumours with low EPR characteristics was significantly lower (*P*<0.001), suggesting passive tumour accumulation of the nanostars *via* the EPR effect. Survival of mice treated with high dose ^177^Lu-labelled star polymers was significantly higher than survival of mice treated with lower therapy doses or control mice (*P*=0.001), demonstrating the utility of the ^177^Lu-labelled star polymers as platforms for endoradiotherapy.

**Conclusion:** Our work highlights the potential of star polymers as probes for the molecular imaging of cancer tissue or for the passive delivery of radionuclides for endoradiotherapy. Their high functionalisability and high tumour accumulation emphasises their versatility as powerful tools for nanomedicine.

## Introduction

Treatment of cancer patients with chemo- and radiotherapeutic agents is generally based on the expression of disease-specific biomarkers that represent cancer stage or disease progression. Moreover, with the emergence of personalised medicine, biomarker status at the tumour site has become increasingly important. Unfortunately, expression levels of biomarkers are usually unknown at initial diagnosis. For visualisation and quantification prior to treatment, molecular imaging probes are commonly based on pharmaceutical analogues of small molecules, peptides or antibodies that target prognostic or predictive biomarkers 1. To assess biomarker status at the genomic level using genetic profiling techniques, the collection of tissue specimens *via* surgery or biopsy is generally required. Although minimally invasive profiling assays based on body fluid-derived specimens have been developed (*i.e.* liquid biopsies), they still suffer from low sensitivity and heterogeneous circulation levels of biomarkers, and translation towards clinical application is still in its early stages 2, 3. The development of theranostic probes that do not rely on biomarker expression would expand therapy options for cancer patients in general and facilitate (neo)adjuvant treatment of inoperable patients. In addition, such probes may bypass the common clinical limitation of acquired resistance against biomarker-based therapies 4, 5.

To achieve accumulation of molecular imaging tracers or radiopharmaceuticals at the tumour site independent of biomarker expression, nanoparticles are the ideal platform. Nanoparticles can passively accumulate at the tumour site *via* the enhanced permeability and retention (EPR) effect often observed in solid tumours, which refers to the phenomenon of extensive angiogenesis, defective vascular architecture, impaired lymphatic drainage and increased expression of proteins associated with vascular permeability 6, 7. In the preclinical setting, the EPR effect has been a widely accepted and proven strategy for the effective delivery of nanoparticles to the tumour site 8, 9. Its utility in a clinical setting is still controversial due to the low overall median tumour accumulation of the current generation of nanoparticles 10. Nevertheless, the majority of nanoparticles currently approved for clinical use (*e.g.* Doxil, Abraxane) rely on passive tumour targeting *via* the EPR effect 8, 10-12. In addition to tumour model, nanoparticle type (organic vs. inorganic), shape (spherical, rod, other), size (hydrodynamic diameter < 100 nm) and targeting strategy (active vs. passive) all contribute to the effectiveness of targeting the tumour and its microenvironment 10.

Polymeric star nanoparticles, also known as nanostars, are a class of macromolecules with a well-defined architecture of linear arms cross-linked at one end to form a central core. Due to their unique design and attractive chemical and physical properties, including their ease of synthesis, low viscosity, compact three-dimensional structure, high functionalisation potential and favourable biological characteristics, star polymers have attracted much interest in recent years as versatile platforms for theranostic applications 13, 14. We have shown that star polymers could be synthesised *via* an 'arm-first' approach, using reversible addition-fragmentation chain-transfer (RAFT) polymerisation 15. Functionalisation of the nanostars with gadolinium(III) (Gd^3+^) as a contrast agent for magnetic resonance imaging (MRI) significantly increased the water proton spin-lattice relaxation rates (*R*_1_), which in turn resulted in enhanced image contrast in *T*_1_-weighted MRI, thus demonstrating their potential as MRI contrast agents 16, 17. In a one-pot synthesis with the radioisotope iodine-125 (^125^I), we further showed that molecular imaging and nuclear medicine could be combined within a single nanostar 18. To further emphasise their theranostic potential, we showed that the nanostars were effectively taken up by MCF-7 breast cancer cells for the endosomal delivery of doxorubicin 19.

In the current study we describe a star polymer in which amines are introduced as a functional handle for later modification with radionuclides, in addition to the Gd^3+^-functionalised monomer units also present in our previous star polymers. Relaxivity measurements at low magnetic field strengths demonstrate that the nanostars exhibit favourable paramagnetic properties, leading to increased longitudinal relaxivity and thus enhanced contrast in *T*_1_-weighted magnetic resonance images. At higher magnetic field strengths, the nanostars display a nearly two-fold increase in the *T*_1_-weighted image contrast compared to the clinical MRI contrast agent Gadovist. To quantify tumour uptake in *in vivo* tumour models, we functionalised the amines on the nanostars with chelators for radiolabelling. Nanostars radiolabelled with positron emitter zirconium-89 (^89^Zr) demonstrate an exceptionally high accumulation at the tumour site, compared to other nanoparticles reported in the literature 10. In a direct comparison, we show that nanostar accumulation in tumours with high vascular permeability is significantly higher than in tumours that exhibit less pronounced EPR characteristics, suggesting passive delivery of the nanostars to the tumour *via* the EPR effect. High tumour accumulation is confirmed using star polymers radiolabelled with beta emitter lutetium-177 (^177^Lu), and we demonstrate the high therapeutic potential of the nanostars.

With regards to tumour accumulation, the star polymers compete with the top range nanoparticles published in current literature 9, 10. Due to their demonstrated chemical and physiological versatility in the current study, the nanostars show great promise as platforms for theranostic applications. Not only would the star polymer scaffolds improve delivery of molecular imaging and (radio)pharmaceutical agents to tumour tissues independent of biomarker expression, but they also offer opportunities for novel imaging technologies, such as multimodal imaging, and machine learning 20, 21.

## Results and Discussion

### Synthesis and Characterisation of the Nanostars

**Figure 1:**
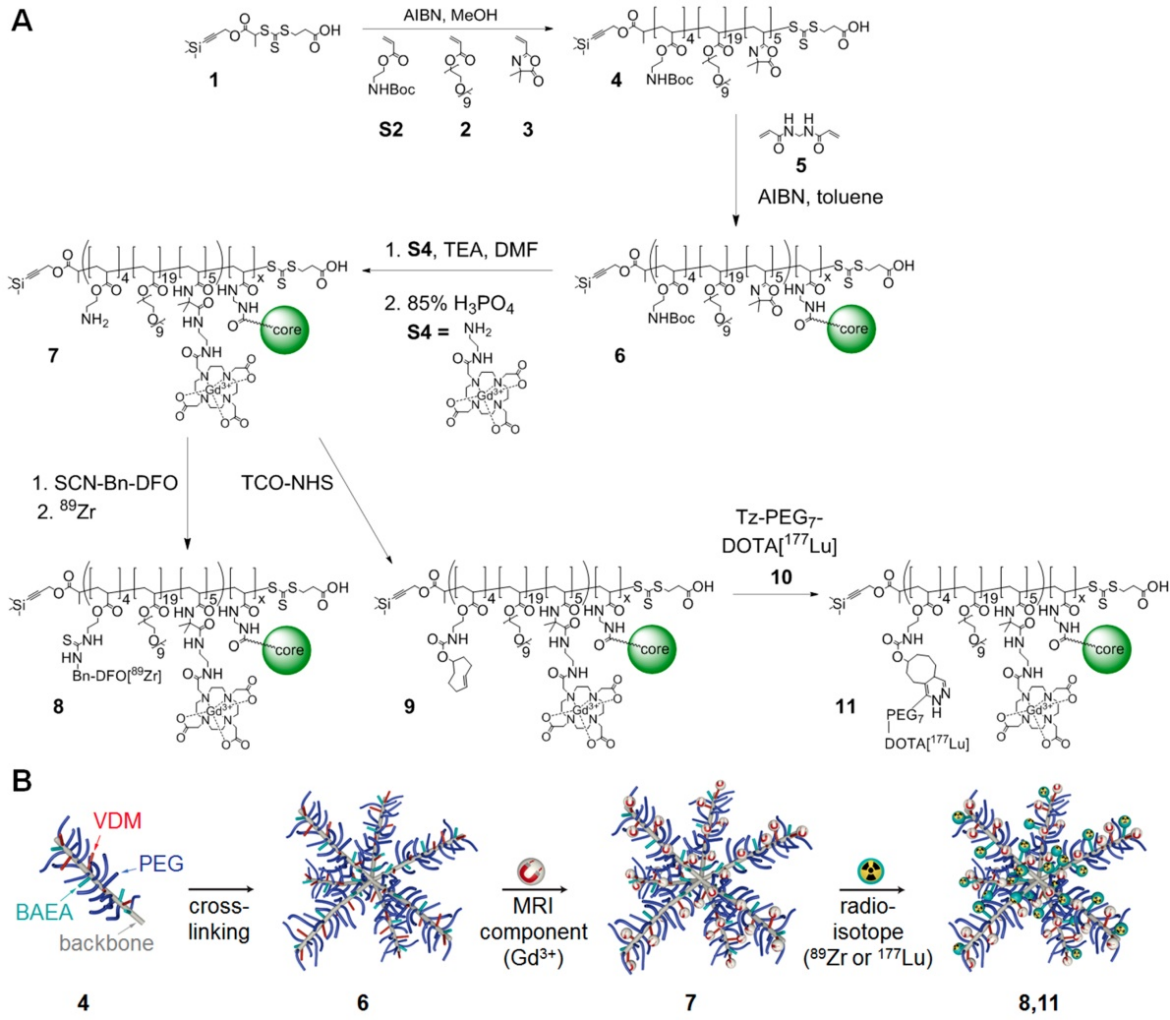
(A) Synthetic route of the star polymer (detailed reaction conditions are provided in the Methods section). (B) Schematic representation of theranostic star polymer synthesis.

**Figure 2:**
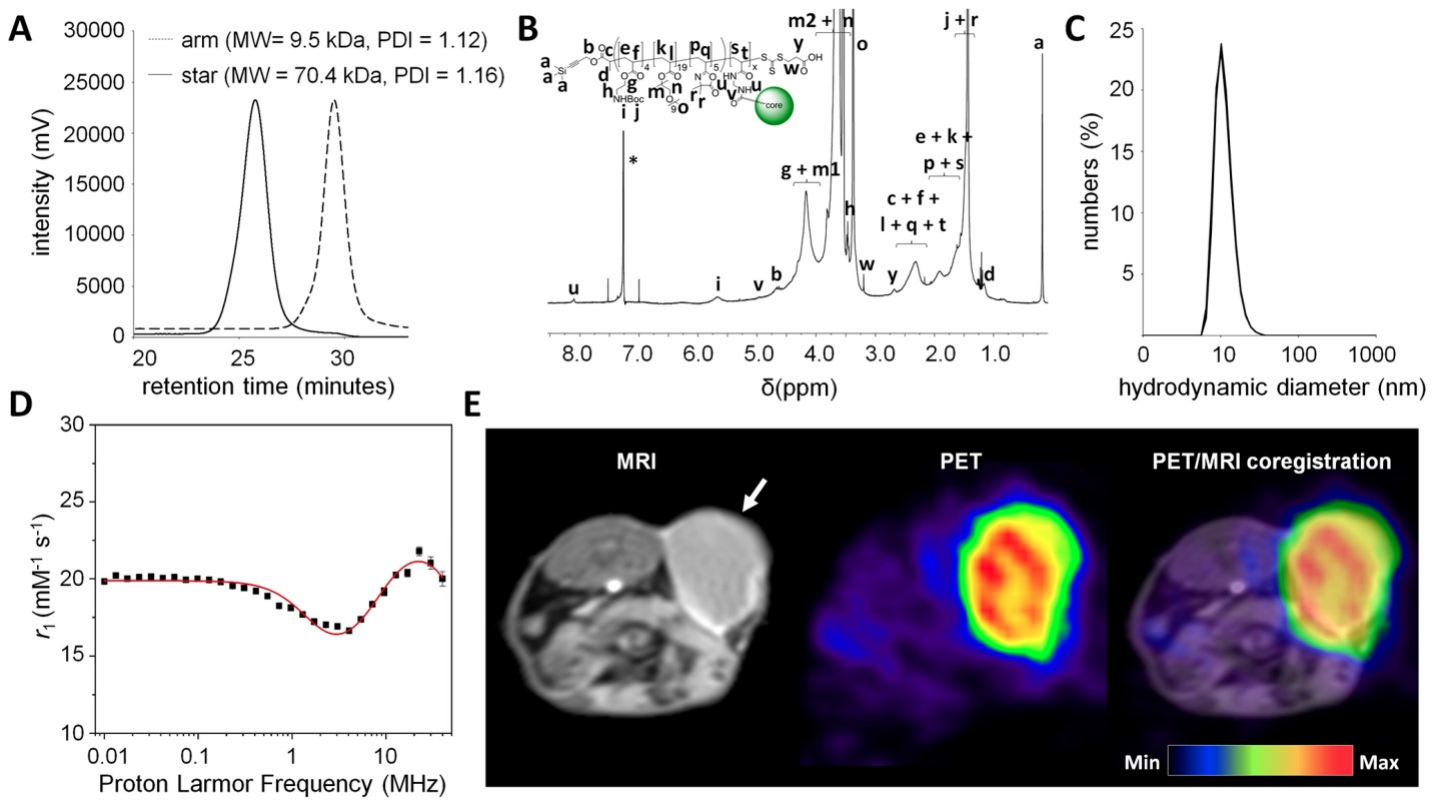
Characterization of the star polymers. (A) GPC traces of p(BAEA-*co*-OEGA-*co*-VDM) linear arm polymer (**4**; MW = 9.5 kDa, PDI = 1.12) and p(BAEA-*co*-OEGA-*co*-VDM) star polymer (**6**; MW = 70.4 kDa, PDI = 1.16). PDI: polydispersity index. (B) ^1^H NMR spectrum of p(BAEA-*co*-OEGA-*co*-VDM) star polymer (**6**) in CDCl_3_ (residual solvent peak indicated by *). (C) Size distribution profile of p(AEA-*co*-OEGA-*co*-[Gd^3+^]VDMD) star polymer (**7**; D_h_ =11 nm), as determined *in triplo* by dynamic light scattering (DLS). D_h_: Number-average hydrodynamic diameter. (D) Model fitting of the nuclear magnetic relaxation dispersion (NMRD) profile of the nanostars obtained at 37°C. The best fit was obtained when using regular inner (IS) and outer (OS) sphere models under standard assumptions, extended to include the contributions from second sphere (SS) water molecules. The NMRD profile was characteristic for a slowly reorienting Gd^3+^ complex (max. relaxivity: 24.4 mM^-1^s^-1^ at 22.6 MHz). (E) Enhanced *T*_1_-weighted contrast was observed in BALB/c mice (n=3) carrying CT26 tumours, 3 days after the injection of nanostar **6** (p(BAEA-*co*-OEGA-*co*-VDM)) (tumour indicated by white arrow). PET images were obtained by co-injection of ^89^Zr-labelled nanostar **8** (p([^89^Zr]Zr-DFO-AEA-*co*-OEGA-*co*-[Gd^3+^]VDMD)).

Nanostars were produced using the arm-first approach *via* RAFT polymerisation (Figure 1) 13, 15. First, linear polymer arms (**4**) were synthesised, consisting on average of 19× oligoethylene glycol methyl ether acrylate (OEGA) monomer units, 5× 2-vinyl-4,4-dimethyl-5-oxazolone (VDM) monomer units and 4× *Boc*-protected aminoethyl acrylate (BAEA) monomer units, as confirmed by ^1^H NMR (Figure S1) 22, 23. In general, a high poly(ethylene glycol) (PEG) content (9× PEG units per OEGA unit) leads to low toxicity of the nanoparticle, as well as a prolonged blood half-life due to its anti-fouling properties 24. Azlactone-based monomer units (VDM) were introduced for functionalisation of the nanostars with DO3A-chelated Gd^3+^, which - due to its paramagnetic properties - enhances image contrast in MRI 25. To allow for additional functionalisation of the nanostars, we introduced multiple Boc-protected amine moieties (BAEA) to the polymer arms. After *Boc*-deprotection of the amines, these functional groups could be conjugated to (radio)therapeutic agents using standard amine-reactive groups such as *N*-hydroxysuccinimide (NHS) esters or thiocyanates (SCN). To ensure that the pharmacokinetic properties of the nanostars were primarily dictated by the PEG component, the combined molecular weight fraction of VDM and BAEA monomer units was kept below 10% 26. Polymer arms (MW = 9.5 kDa) were obtained with high purity (>99%) and good polydispersity index (PDI = 1.12) (Figures 2A and S1). Polymerisation kinetics indicated that VDM monomer was preferentially consumed over both OEGA and BAEA monomers (Figure S1), resulting in a decreasing gradient of VDM from the exterior of the polymer arms towards the interior. This gradient would benefit MRI contrast enhancement, since chelated Gd^3+^ atoms at the exterior of nanostars would be more readily accessible to exchanging water molecules 17. The linear arms were then cross-linked at one end *via* chain extension with a difunctional crosslinking monomer (*N*,*N*′-methylenebis(acrylamide)), forming well-defined star polymer **6** (p(BAEA-*co*-OEGA-*co*-VDM); MW = 70.4 kDa, PDI = 1.16) with on average 7 to 8 arms per nanostar, as calculated from the average molecular weight increase measured by gel permeation chromatography (GPC) in comparison to the average molecular weight of a single linear polymer arm (Figure 2A). Unreacted polymer arms were successfully removed by precipitation, yielding pure product **6** (>99%) with a hydrodynamic diameter (D_h_) of 13 nm (Figures 2B and S2).

The RAFT agent used for polymerisation was equipped with a trimethylsilyl-protected alkyne, offering additional functionalisation opportunities at the outer periphery of the nanostar using traditional alkyne-azide click chemistry 16. To provide proof-of-concept, nanostar **6** was functionalised with fluorescent dye Fluor 488 (excitation wavelength = 488 nm) on the RAFT agent. After removal of the trimethylsilyl protecting group, indicated by disappearance of its ^1^H NMR peak at δ = 0.06 ppm (Figure S3A), azide-functionalised Fluor 488 was conjugated to the alkyne on the RAFT agent *via* copper-click chemistry. GPC demonstrated that product with an excitation wavelength of 488 nm eluted at the expected elution time of the star polymer, suggesting that conjugation of the dye to the nanostar was successful (Figure S3B). Functionalisation of the nanostars with Fluor 488 shows that the alkynes can be conjugated to chemically or physiologically active agents, such as fluorescent dyes or drugs like doxorubicin, emphasising the versatility of the nanostars as platforms for theranostics 19. Since the alkyne-deprotection conditions used here are not optimal for the hydrolysis-sensitive VDM monomers, functionalisation of VDM is preferred prior to functionalisation of the alkynes. This experiment, however, provides proof-of-concept of the functionalisation potential of the RAFT agent. For simplicity, further experiments were performed using the trimethylsilyl-protected star polymers.

Gd^3+^ was introduced to nanostar **6** by conjugating [Gd^3+^]DO3A to its VDM monomer units. To this end, 1-(5-amino-3-aza-2-oxypentyl)-4,7,10-tris(*tert*-butoxycarbonylmethyl)-1,4,7,10-tetraazacyclododecane (DO3A-*t*Bu-NH_2_) was acid-deprotected and loaded with Gd^3+^. Resulting product **S4** (2-aminoethyl-mono-amide-[Gd^3+^]DO3A) was reacted to the VDM monomer units in star polymer **6**, followed by *Boc-*deprotection of the amine moieties under standard acidic conditions. The presence of Gd^3+^ in nanostar **7** (p(AEA-*co*-OEGA-*co*-[Gd^3+^]VDMD); D_h_ = 11 nm) led to considerable peak broadening in the ^1^H NMR spectrum, due to Gd^3+^-mediated shortening of *T_2_* on nearby protons, suggesting successful conjugation (Figures 2C, S4 and S5). Inductively coupled plasma (ICP) spectrometry showed that star polymer **7** contained 10-11 Gd^3+^ atoms per molecule.

### Nanostars Enhance MRI Contrast by Shortening Water Proton *T*_1_ Relaxation Times

To assess the ability of the nanostars to enhance MRI contrast, the magnetic properties of nanostar **7** (p(AEA-*co*-OEGA-*co*-[Gd^3+^]VDMD) were measured at different magnetic field strengths. Its longitudinal relaxivity at lower field strengths (0.24 mT - 0.94 T; 0.01 - 40 MHz) was measured by ^1^H NMR relaxometry, generating a nuclear magnetic resonance dispersion (NMRD) profile characteristic of a slowly reorienting Gd^3+^ complex (Figure 2D) 16, 27. Maximal relaxivity at 37°C was 24.4 mM^-1^s^-1^ at 22.6 MHz, which is higher than for our previously reported star polymers and, more importantly, over 6-fold higher than reported for clinically used contrast agents such as Gadovist at similar field strength (3.7 mM^-1^s^-1^ at 20 MHz) 16-19, 28-30.

The relaxivity of paramagnetic macromolecular nanostructures is defined by their molecular reorientation, water exchange and electronic relaxation properties 27, 31, 32. Therefore, we characterised the paramagnetic properties of the nanostars by deducing standard relaxation parameters from the ^1^H NMRD profile at 310 K, including water residence time (*τ_m_*), reorientational correlation time (*τ_R_*) and electronic relaxation correlation time (*τ*_v_). For quantitative assessment of these parameters, the ^1^H NMRD profile was fit using regular inner (Solomon-Bloembergen-Morgan) and outer (Hwang and Freed) sphere models under standard assumptions, extended to include contributions from second sphere water molecules (Figure 2D 31-38. For estimation of the standard relaxation parameters, the following parameters were fixed during the fitting procedure: distance between the Gd^3+^ ion and the coordinated inner sphere water proton (*r*_Gd-H_ = 3.1 Å), number of inner sphere water molecules coordinated to Gd^3+^ (*q* = 1), water self-diffusion coefficient (*D* = 2.9 × 10^-9^ m^2^ s^-1^), distance of closest approach between Gd^3+^ ion and neighbouring outer sphere water proton (*d* = 3.6 Å), distance between Gd^3+^ ion and second sphere water proton (*r*_ss_ = 3.6 Å) and the hydration time of the second sphere water molecules (*τ*_ss_ = 65 ps) 17, 39, 40. The parameters resulting from the most optimal fit are summarised in Table 1.

The water residence time (*τ*_M_ = 853 ± 43 ns) was typical of a star polymer functionalised with amide-based Gd^3+^-complexing groups 16, 17. As expected, the residence time of the water molecules coordinated to the nanostar was relatively high compared to their reported water residence times in the presence of clinically applied small molecule contrast agents 30. A short water residence time implies fast water exchange and increased relaxivity, hence enhanced contrast on MR images, which is much easier to achieve for small molecules than for macromolecules. These data suggest that MRI contrast of the nanostars might be improved by increasing the water exchange rate of the nanostars, for example by adjusting the monomer ratios of the polymer arms 17. Alternatively, substitution of the linker used to conjugate [Gd^3+^]DO3A to the nanostars by an amide-free analogue may result in a 2 to 8 times faster water exchange rate 41.

The increased relaxivity of the nanostars at these field strengths compared to small molecule contrast agents is largely dictated by their long reorientational correlation time (*τ*_R_ = 3.9 ± 1.4 ns), which is several orders of magnitude higher than the reorientational correlation time of clinical contrast agents 30. A long reorientational correlation time suggests restricted internal motion of the Gd^3+^ complex, which leads to enhanced MRI contrast, although it must be noted that the positive effect of the slower tumbling rate on the relaxivity tends to decrease with increasing field strengths 42.

The electronic relaxation time at zero field strength (*τ*_so_ = 196 ± 11 ps) and electronic relaxation correlation time (*τ*_v_ = 42 ± 2 ps) were in the same range as electronic relaxation parameters previously reported for star polymers and were higher than those reported for most clinical small molecule contrast agents 17, 30. At low magnetic field strengths, the effect of electronic relaxation contributes to the increased relaxivity of the nanostars compared to small molecule MRI contrast agents commonly used in the clinic 30.

The paramagnetic properties of the star polymers were visualised at 3 T using a dilution series of the star polymer in HEPES buffer (pH 7.4). At higher concentrations, an increased spin-lattice relaxation rate (*R*_1_) was observed (Figure S6), resulting in significant *T*_1_-weighted contrast enhancement (Figure S7). Similarly, at 7 T, the nanostars significantly decreased the water proton *T*_1_ relaxation time (*T*_1_ = 0.60 s) compared to that of the small molecule contrast agent Gadovist (*T*_1_ = 1.0 s) at equimolar Gd^3+^ concentrations, which denotes a *T*_1_ shortening of 1.7× (Figure S7).

To demonstrate the applicability of the nanostars as *T*_1_-weighted MRI contrast agents *in vivo*, MR images were acquired 3 days after the injection of 3 mg or 10 mg of nanostar **6** (p(BAEA-*co*-OEGA-*co*-VDM)) in mice (*n* = 3-5) engrafted with subcutaneous CT26 tumours. After the injection of 3 mg nanostar, some *T*_1_-weighted contrast enhancement was indeed observed in the tumour tissue (Figure S8A). After the injection of 10 mg nanostar, tumours could clearly be delineated due to the considerable contrast enhancement in the tumour, which was significantly higher than the tumour contrast prior injection (*P* = 0.013; Figures 2E and S8B). At this high dose (10 mg), however, some haematological toxicity was observed, leading to the death of one of the investigated mice. This toxicity was likely caused by the high concentration of nanostars accumulating in tissues that play an important role in red blood cell metabolism, such as liver and spleen, leading to a gradual - but lethal - decrease in red blood cell counts and haematocrit values (Figure S8C). Such toxic effects were not observed at a lower nanostar dose (3 mg) and, therefore, further evaluation of the optimal MR imaging dose is required.

These data provide proof-of-concept that - after further dose optimization - the paramagnetic characteristics of the star polymers would allow for their application as *T*_1_ MRI contrast agents in a (pre)clinical setting.

### Nanostars Highly Accumulate in Tumours *via* EPR Effect

To quantify tumour accumulation of the star polymers in tumour tissue, star polymer **7** (p(AEA-*co*-OEGA-*co*-[Gd^3+^]VDMD) was functionalised with deferoxamine (DFO) for radiolabelling of the nanostars with ^89^Zr. Successful synthesis of star polymer p(DFO-AEA-*co*-OEGA-*co*-[Gd^3+^]VDMD) (D_h_ = 13 nm) was confirmed by the appearance of DFO-specific peaks at δ = 7.2 - 8.2 ppm in the ^1^H NMR spectrum (Figure S9). Radiolabelling of p(DFO-AEA-*co*-OEGA-*co*-[Gd^3+^]VDMD with ^89^Zr^4+^ was achieved with high radiochemical yield (>99%) and purity (>99%) and good molar activity (>290 GBq/µmol) (Figure S10). *In vitro* stability assays demonstrated that the resulting star polymer **8** (p([^89^Zr]Zr-DFO-AEA-*co*-OEGA-*co*-[Gd^3+^]VDMD)) was stable to decomposition in human serum over time (Figure S11).

**Figure 3:**
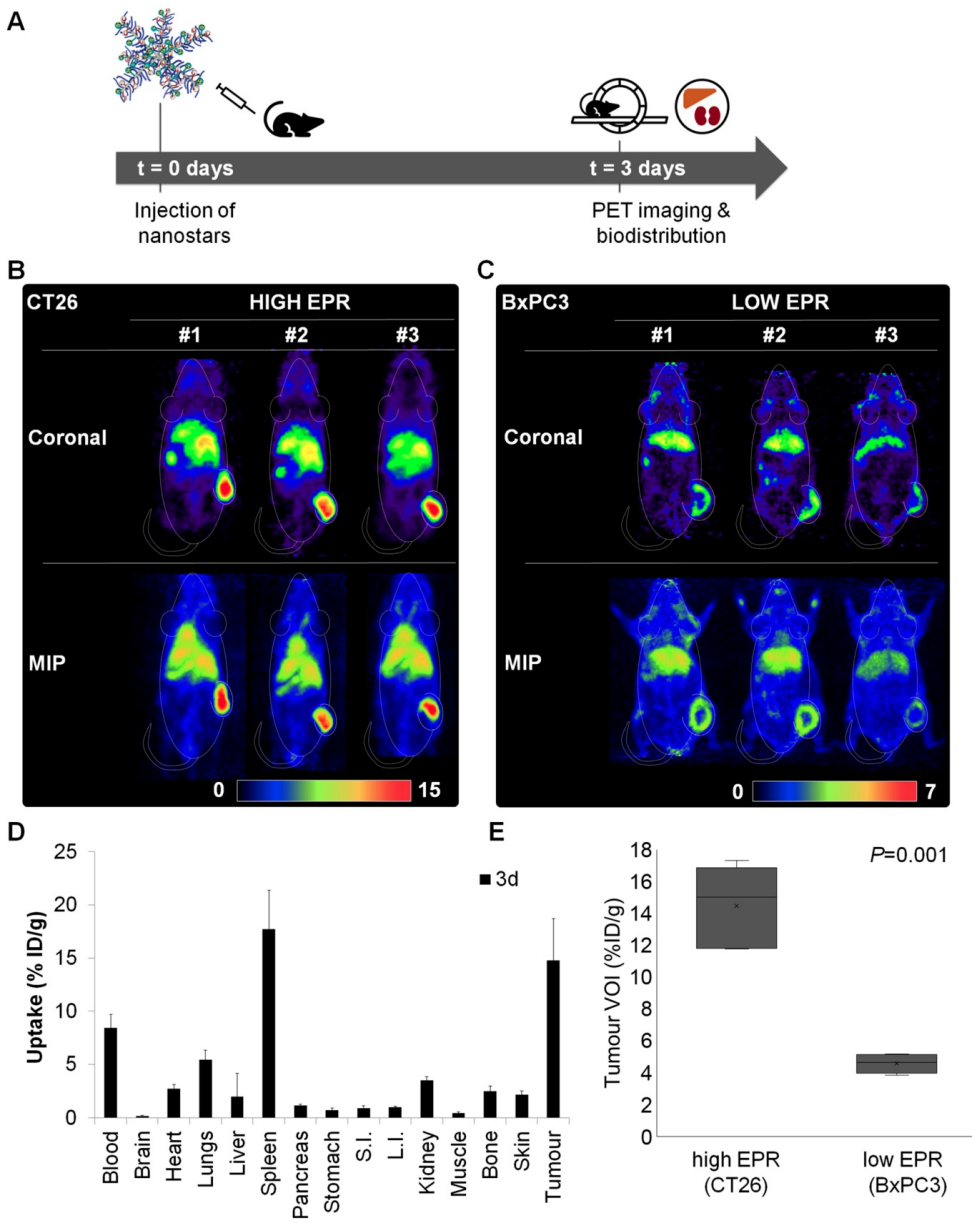
*In vivo* quantification and positron emission tomography (PET) imaging of ^89^Zr-labelled nanostar **8** (p([^89^Zr]Zr-DFO-AEA-*co*-OEGA-*co*-[Gd^3+^]VDMD)). (A) Uptake study scheme of ^89^Zr-labelled nanostars in BALB/c mice, isografted with CT26 colon cancer (high EPR) or xenografted with BxPC3 pancreatic cancer (low EPR) cells. (B-C) Representative coronal and maximum intensity projection (MIP) PET images of BALB/c mice (n=5 per study) carrying CT26 or BxPC3 tumours, 3 days after injection of ^89^Zr-labelled nanostars (~10 MBq, MA = 64 GBq/µmol). High accumulation of the ^89^Zr-labelled nanostars was observed in CT26 isografts, whereas low and mainly peripheral accumulation was observed in BxPC3 xenografts, indicating passive tumour uptake *via* the EPR effect. Scale bars are in %ID/g. (D) Biodistribution profile 3 days after injection of ^89^Zr-labelled nanostars in BALB/c mice isografted with CT26 colon cancer cells. Highest accumulation was observed in tumour tissue and spleen. S.I.: small intestine, L.I.: large intestine. (E) Comparison of maximum uptake values in volumes of interest (VOIs) of CT26 and BxPC3 tumours. Uptake of ^89^Zr-labelled in CT26 (high EPR) isografts was significantly higher than uptake in BxPC3 (low EPR) xenografts, as determined by the independent-samples t-test (*P*=0.001).

To demonstrate that uptake of the ^89^Zr-labelled nanostars was dominated by passive targeting *via* the EPR effect, tumour accumulation was compared between tumour models with highly leaky vasculature (CT26 colon cancer isografts; “high EPR”) and poorly leaky vasculature (BxPC3 pancreatic cancer xenografts; “low EPR”) 43. Mice (*n* = 5) engrafted with subcutaneous CT26 or BxPC3 tumours were administered 10 MBq of nanostar **8** (MA = 64 GBq/µmol). For visualisation of tumour accumulation, PET images were acquired 3 days after injection of the ^89^Zr-labelled nanostars (Figure 3A). The radiolabelled nanostars demonstrated a very clear delineation of tumours with high EPR characteristics (Figures 2E and 3B). Although peripheral tumour accumulation was also observed in tumours with low EPR characteristics, contrast relative to other tissues was less pronounced in low EPR tumours (Figure 3C).

The high uptake in tumours with high EPR characteristics was confirmed by *ex vivo* biodistribution experiments, demonstrating a tumour accumulation of 14.8 ± 4.0 %ID/g after 3 days (Figure 3D). Considerably lower tumour uptake values have been reported for the current generation of nanoparticles, emphasising the remarkably high tumour accumulation observed for the star polymers in this study 10. This could be explained by the size, composition and flexibility of the nanostars 44, 45. First, the large size of the nanostars prevents their renal clearance, but enables their accumulation at the tumour site through the leaky vasculature associated with the EPR effect. Second, the primary component of the nanostars is PEG, which extends their blood circulation time and, as such, prolongs their exposure to the tumour. Third, the stereochemical flexibility of the nanostars facilitates their deep penetration into the tumour tissue, contributing to the high uptake levels at the tumour site. Higher tumour uptake values for passively targeting nanoparticles have only been reported for a few inorganic and fewer organic nanoparticles 9, 10. However, inconveniently high liver and spleen uptake values were also reported for those nanoparticles, in most cases significantly higher than the tumour uptake values 9. In the current study, the non-target organ with the highest concentration of ^89^Zr-labelled nanostars was the spleen, with an uptake value that was similar to uptake in the tumour (17.7 ± 3.7 %ID/g). Besides accumulation of the nanostars in the blood circulation (8.4 ± 1.3 %ID/g), all other organs displayed uptake values of less than 5.5 %ID/g.

The peripheral tumour accumulation observed in the BxPC3 xenografts highlights the poor penetration of the ^89^Zr-labelled nanostars into the low EPR tumours (Figure 3C). That is, pancreatic tumours are notorious for their dense extracellular matrix, restricting penetration of (nano)therapeutic agents in general 46, 47. Uptake differences between low and high EPR tumours were quantified by comparing the maximal uptake values in three-dimensional volumes of interest (VOIs) obtained from the PET images of the low and high EPR tumour models (Figure 3E). Evidently, nanostar uptake values in tumours with high EPR characteristics were significantly higher than in tumours with low EPR characteristics (*P*<0.001), suggesting that uptake of the nanostars in tumour tissue is mainly dictated by passive accumulation *via* the EPR effect.

Although the EPR effect has been a mainstay delivery strategy for nanoparticles in the preclinical setting, its clinical utility remains debated 8. Clinically approved nanoparticles that rely on passive tumour targeting have only shown limited efficacy due to heterogeneity across patients and cancer types, which has contributed to the questionable reputation of nanoparticles in clinic 8. To account for the apparent heterogeneity and to expand our understanding of the EPR effect in human cancers, molecular imaging agents that can quantify and visualise the EPR effect in patients are needed. The radiolabelled nanostars may benefit cancer imaging and treatment by selecting patients for nanomedicine that demonstrate enhanced permeability and retention of the nanostars, with the aim to increase the efficacy of currently approved nanotherapeutics. The combination of a paramagnetic component (Gd^3+^) and a radionuclide (^89^Zr^4+^) within a single nanoparticle would also allow for multimodal imaging, for example using emerging hybrid imaging techniques such as simultaneous PET/MRI imaging, as well as facilitate the spatial co-registration of multimodal images in machine learning 8, 48. Additionally, the high accumulation of the nanostars in high EPR tumours suggest that they could be used as platforms for the treatment of solid tumours with leaky vasculature, after their functionalisation with (radio)therapeutic agents, for example when patients appear inoperable due to an unfavourable anatomical location of the tumour or when patients have become resistant to standard antibody-based therapies 19, 49.

### Endoradiotherapy with Nanostars Leads to Prolonged Survival

To assess the endoradiotherapeutic potential of the nanostars, star polymer **7** (p(AEA-*co*-OEGA-*co*-[Gd^3+^]VDMD) was functionalised with *trans*-cyclooctene (TCO) to enable radiolabelling of the nanostars with a ^177^Lu-labelled DOTA-functionalised tetrazine (Tz). We have previously used a similar two-step approach for the radiolabelling of monoclonal antibodies with actinium-225 (^225^Ac) 50. Here, this labeling approach was chosen to prevent metal exchange of Gd^3+^ and ^177^Lu^3+^ during the radiolabelling procedure, which are both complexed by a structurally similar chelator (DO3A and DOTA, respectively). Successful synthesis of star polymer **9** (p(TCO-AEA-*co*-OEGA-*co*-[Gd^3+^]VDMD); D_h_ = 12 nm) was confirmed by the appearance of TCO-specific peaks at δ = 5.5 ppm and δ = 1.0 - 2.0 ppm in the ^1^H NMR spectrum (Figure S12). Tetrazine-poly(ethylene glycol)_7_-DOTA (**10**, Tz-PEG_7_-DOTA) was radiolabelled with ^177^Lu^3+^ and reacted to star polymer **9** to obtain star polymer **11** (p([^177^Lu]Lu-DPAEA-*co*-OEGA-*co*-[Gd^3+^]VDMD)) with high radiochemical yield (87%) and purity (>99%) and high molar activity (615 GBq/µmol) (Figure S13).

**Figure 4:**
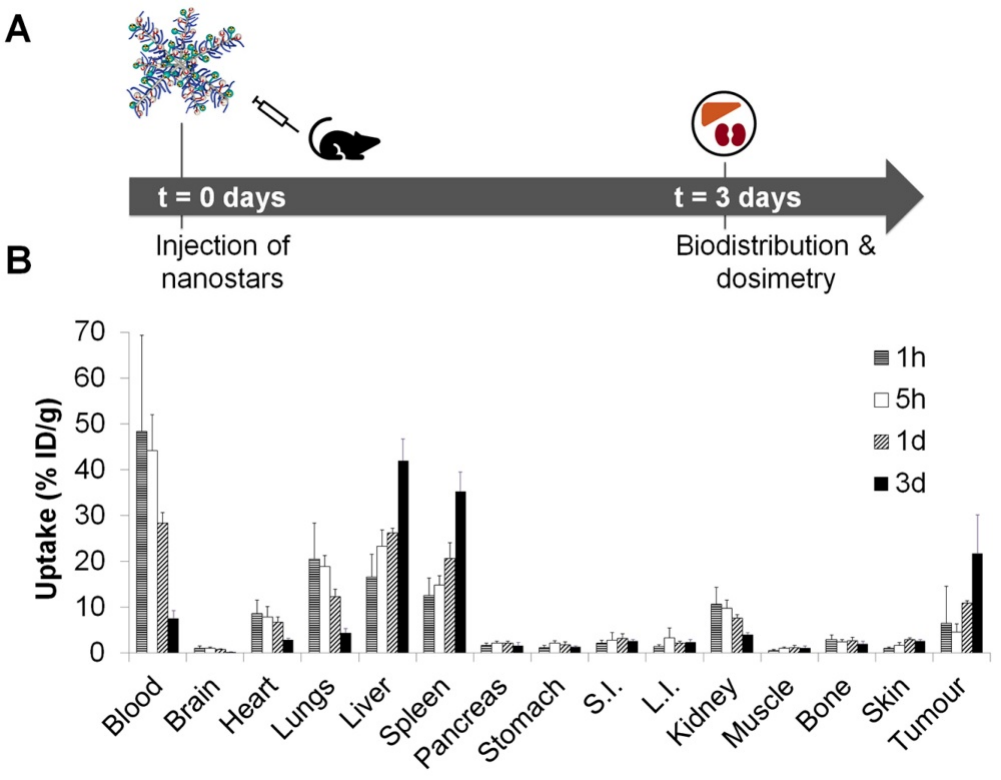
*In vivo* quantification of ^177^Lu-labelled nanostar **11** (p([^177^Lu]Lu-DPAEA-*co*-OEGA-*co*-[Gd^3+^]VDMD)). (A) Uptake study scheme of ^177^Lu-labelled nanostars in BALB/c mice, isografted with CT26 colon cancer cells. (B) Biodistribution profile of ^177^Lu-labelled nanostars (~0.8 MBq, MA = ~5 GBq/µmol) up to 3 days after injection in BALB/c mice isografted with CT26 colon cancer cells. Highest tumour accumulation was observed after 3 days, as well as accumulation in liver and spleen. S.I.: small intestine, L.I.: large intestine.

The activity distribution of the ^177^Lu-labelled nanostars was measured in mice (*n* = 4 per timepoint), bearing subcutaneous CT26 isografts (*i.e.* high EPR tumours). Biodistribution profiles were obtained at different time points after the injection of 0.8 MBq of nanostar **11** (MA = 5 GBq/µmol) (Figure 4A). The biodistribution profile of the ^177^Lu-labelled nanostars after 3 days demonstrated a remarkably high uptake in tumour tissue (21.7 ± 8.4 %ID/g) (Figure 4B), which was even higher than the observed uptake of the ^89^Zr-labelled nanostars (Figure 3D).

These data confirmed the clearance of the nanostars from the blood majorly *via* liver and spleen, after an apparent partial initial clearance via the kidneys. Although the high splenic uptake (35.3 ± 4.2 %ID/g) was observed for the ^89^Zr-labelled nanostars as well, the ^177^Lu-labelled nanostars also displayed high accumulation in liver tissue (42.0 ± 4.7 %ID/g). This discrepancy may be explained by changes in molecular weight or surface charge of the star polymers after reaction with [^177^Lu]Lu-Tz-PEG_7_-DOTA, rather than accumulation of detached [^177^Lu]Lu-Tz-PEG_7_-DOTA or free ^177^Lu^3+^ after demetallation from the DOTA chelators. That is, clearance of unbound [^177^Lu]Lu-Tz-PEG_7_-DOTA is likely to proceed *via* the urinary tract instead of the hepatobiliary tract and free ^177^Lu^3+^ tends to accumulate in bone 51. Except for accumulation in the blood (7.5 ± 1.8 %ID/g), uptake of the ^177^Lu-labelled nanostars in all other organs was less than 5.0 %ID/g.

Accumulation of the ^177^Lu-labelled nanostars in tumour tissue seemed to decrease after 3 days (Figure S14), possibly implying washout of the nanostars from the tumour over time. More likely, however, the seemingly rapid decrease of tumour accumulation at later time points is an artefact of the tumour model used in this study. Namely, the impaired angiogenesis and leaky vasculature of CT26 tumours is inherently caused by rapid growth of the tumour mass 6, 43. In general, for a fair comparison of activity concentrations between tissues, tissue mass and tissue-to-tissue ratios are assumed to remain reasonably consistent over time. In the fast-growing tumour model applied here, however, the tumour mass increased six- to eight-fold over the course of the 14-day ^177^Lu-therapy study (Figure S15A), causing a rapid decrease in uptake values per tumour mass at later time points. Ignoring the weight component from the biodistribution calculations by analysing tumour uptake in %ID instead of %ID/g (Figure S15B), a much less pronounced washout effect is observed, as is indicated by the shallower slope of the uptake curve in %ID after 3 days. These data suggest that the measured tumour uptake values at 7 days and 14 days are an underestimation of the actual nanostar uptake and retention in the tumour, compared to the clinical situation in which tumour growth is generally slower.

Based on the full biodistribution data of the ^177^Lu-labelled nanostars (0-14 days), mean absorbed organ doses in mice were calculated, as well as the therapeutic index per organ, which is defined as the ratio of the radiation-absorbed dose in the tumour divided by the dose in a (radiosensitive) tissue (Table 2) 52. Mean absorbed organ doses in mice were calculated based on trapezoidal integration of the time-activity curves obtained from the biodistribution data (Figure S16) and demonstrated low to moderate therapeutic indices for half of the investigated organs. It must be noted, however, that the values displayed in Table 2 are likely an underestimation of the actual therapeutic indices, since tumour uptake values are based on time-activity curves generated from a fast-growing tumour model, which gives an artificial decrease of ^177^Lu-labelled nanostar accumulation at later time points.

**Figure 5:**
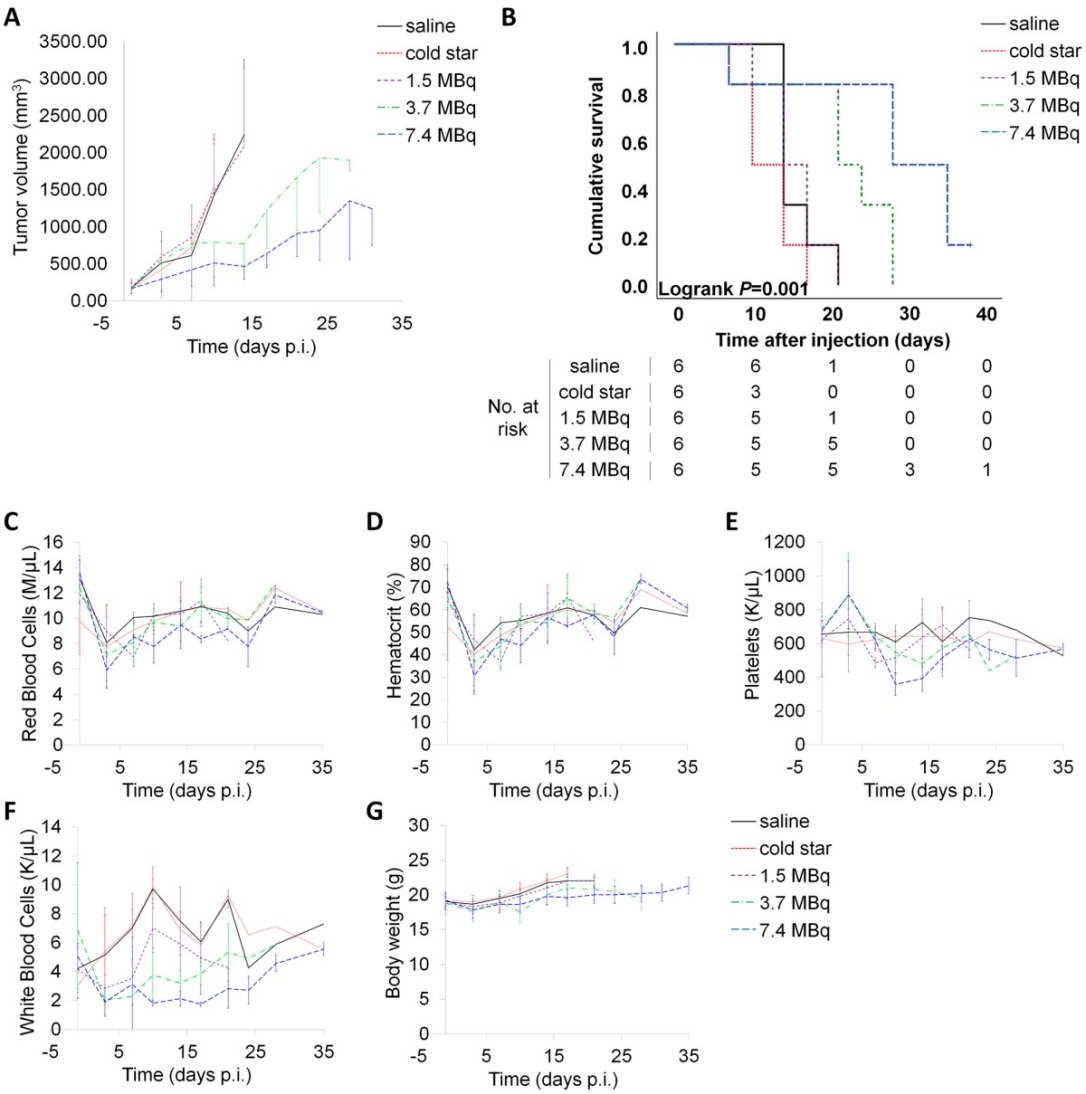
Therapy studies with ^177^Lu-labelled nanostars in BALB/c mice isografted with CT26 colon cancer cells. Mice were treated with 1.5 MBq, 3.7 MBq or 7.4 MBq of p([^177^Lu]Lu-DPAEA-*co*-OEGA-*co*-[Gd^3+^]VDMD) star polymer (**11**). Mice in the two control groups were injected either with vehicle (0.9% sterile saline) or non-radioactive nanostar **6** (p(BAEA-*co*-OEGA-*co*-VDM); 23 µg per mouse). (A) Tumour volumes increased rapidly for mice in the two control groups and the low dose therapy cohort (1.5 MBq). Tumour volumes increased considerably slower for mice treated with medium (3.7 MBq) and high doses (7.4 MBq). (B) The cumulative survival of mice in each cohort increased significantly with increasing therapy dose, demonstrating the therapeutic potential of the ^177^Lu-labelled nanostars. The haematological toxicity of treatment with ^177^Lu-labelled nanostars was assessed by measuring alterations in (C) red blood cell counts, (D) haematocrit values, (E) platelet counts, and (F) white blood cell counts. Further, systemic toxicity was monitored by measuring signs of lethargy, loss of appetite and (G) body weight.

Based on the dosimetry data, maximal administered activities for the therapy study were guided by normal organ radiation absorbed dose tolerances, leading to selection of the following activities for preclinical dose escalation studies: 1.5 MBq (40 µCi), 3.7 MBq (100 µCi) and 7.4 MBq (200 µCi) 52, 53. The majority of nanoparticle-based therapies, as well as most antibody-based therapies, rely on clearance of the (radio)therapeutic agent *via* regular clearance organs, including the liver and spleen. Indeed, those organs typically demonstrate high uptake of the (radio)therapeutic agents, however, they can also tolerate relatively high absorbed doses (>3000 cGy for liver, >1500 cGy for spleen) 51, 53. Commonly, the dose-limiting tissue for radionuclide therapy is the red marrow, which has a maximum tolerated dose of approximately 150 cGy 52. At a therapeutic administered activity of 3.7 MBq, the estimated absorbed doses to liver, spleen and bone would be 2540 cGy, 1680 cGy and 125 cGy, respectively (Table S1), which are close to or below the maximum tolerated doses for these organs. Therefore, mice bearing subcutaneous CT26 isografts (*i.e.* high EPR tumours) were injected with low dose (1.5 MBq), medium dose (3.7 MBq) or high dose (7.4 MBq) of p([^177^Lu]Lu-DPAEA-*co*-OEGA-*co*-[Gd^3+^]VDMD) star polymer (**11**), after their randomization into five cohorts (*n* = 8 per cohort) with approximately equal tumour volume distributions. Mice in the control groups were injected either with vehicle (0.9% sterile saline) or non-radioactive nanostar **6** (p(BAEA-*co*-OEGA-*co*-VDM). The two mice with the smallest and largest tumours were excluded from each cohort to minimize bias due to outliers, effectively resulting in *n* = 6 mice per cohort. Notably, tumour growth was considerably impaired with increasing therapy doses (Figure 5A). No significant differences in mean tumour volume were observed between the control cohorts and the low therapy dose (1.5 MBq) cohort. Consequently, mice treated with the high dose of ^177^Lu-labelled nanostar (7.4 MBq; median survival = 35 days) demonstrated a significantly prolonged survival (*P* = 0.001) compared to mice treated with the medium therapy dose (3.7 MBq; median survival = 24 days), mice treated with the low therapy dose (1.5 MBq; median survival = 17 days) and mice in the control groups (median survival = 14 days for both groups) (Figure 5B). Haematoanalysis (*n* = 3 per cohort) demonstrated a marked decrease in white blood cell counts for all mice treated with the ^177^Lu-labelled nanostars (Figures 5C-F). For mice in the low therapy dose (1.5 MBq) cohort, white blood cell counts recovered to their initial levels within one week. Mice in the medium (3.7 MBq) and high (7.4 MBq) therapy cohorts recovered their original white blood cell values approximately 28 days after injection. Such decreases in white blood cell values are not uncommon for endoradiotherapeutic approaches and are generally counteracted in the clinical setting by the administration of Granulocyte Colony Stimulating Factor (G-CSF) 54, 55. No significant alterations were observed for the other blood markers, nor did the mice show any outward signs of toxicity, such as lethargy, loss of appetite or decreasing body weight (Figure 5G). In view of these results, endoradiotherapy with ^177^Lu-labelled nanostars is a promising novel treatment strategy for patients with tumours with demonstrated EPR characteristics.

**Figure 6:**
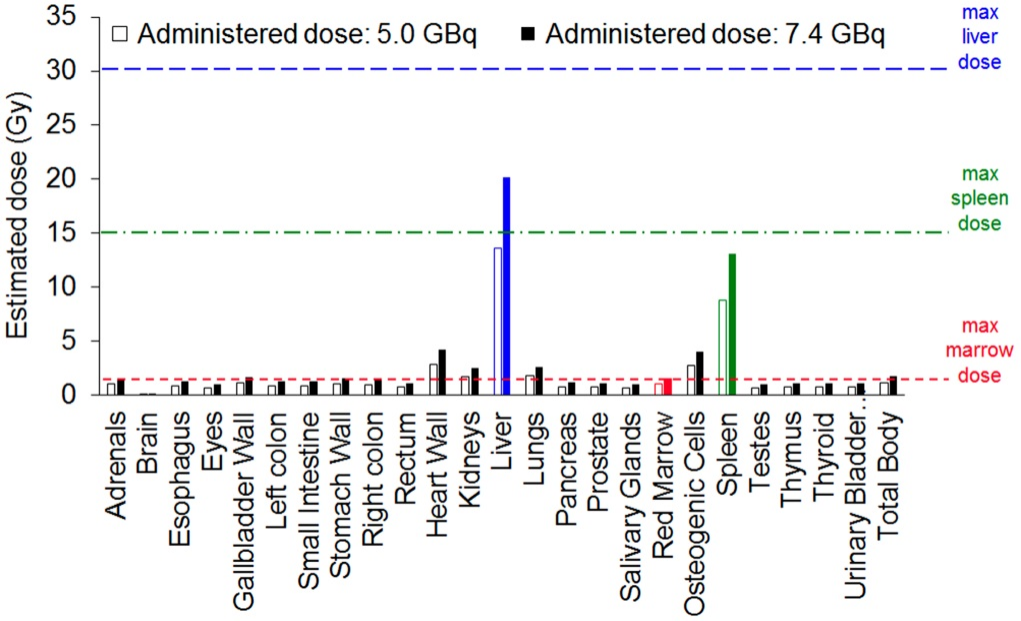
Absorbed dose estimations for the ICRP89 adult male model, calculated from biodistribution data of the ^177^Lu-labelled nanostars in BALB/c mice isografted with CT26 colon cancer cells. Absorbed doses are estimated for administered theoretical doses of 5.0 GBq and 7.4 GBq. Maximum tolerated doses are indicated for liver (blue), spleen (green) and red marrow (red).

To translate dosimetry predictions from mouse to human, mean absorbed organ doses were estimated for the ICRP89 adult male model within the FDA-approved dosimetry software OLINDA 2.0 (Figure 6 and Table S2) 56. In clinical trials with ^177^Lu-labelled monoclonal antibodies, patients are generally administered radioactive doses of ~7.4 GBq (200 mCi) 51. At this dose, the ^177^Lu-labelled nanostars would deliver an estimated dose to red marrow (1.55 Gy) and spleen (13.10 Gy) close to their maximum tolerated dose (1.5 Gy and 15 Gy, respectively). As it is difficult to accurately predict therapeutic response in humans from mouse data, optimisation may be needed to determine at which dose the ^177^Lu-labelled nanostars would demonstrate maximal therapeutic effect with minimal toxicity. Potentially, fractionated administration of lower single doses of ^177^Lu-labelled nanostar would increase efficacy without significant side-effects 57.

## Conclusions

In the current study, we developed star polymers in which multimodality molecular imaging is combined with endoradiotherapy, demonstrating the versatility of nanostars as platforms for theranostics. The star polymers were synthesised *via* 'arm-first' RAFT polymerisation with good polydispersity index and high functionalisation potential, due to the introduction of easily functionalisable amine moieties to the polymer arms and the addition of a functionalisable alkyne at the end of each arm. The presence of Gd^3+^ in the polymer arms led to a more than six-fold improvement of the relaxivity at lower magnetic field strengths, compared to the clinically used contrast agent Gadovist, and demonstrated an almost two-fold enhancement of *T*_1_-weighted image contrast at higher, clinically relevant magnetic field strengths. Functionalisation of the amines with [^89^Zr]Zr-DFO showed that the nanostars accumulated remarkably well in tumours with established EPR properties and significantly less in tumours with less pronounced EPR characteristics, which was confirmed by measuring the tumour uptake of [^177^Lu]Lu-DOTA-functionalised star polymers. The high tumour accumulation of the nanostars (14.8 - 21.7 %ID/g) in tumours with high EPR characteristics suggested that the nanostars are passively taken up in tumour tissue *via* the EPR effect. Moreover, tumour uptake of the nanostars was much higher than has been reported for the majority of nanoparticles elsewhere. The endoradiotherapeutic potential of the nanostars was demonstrated in mice and extrapolated from the mouse experiments to a human model, estimating that at standard injected doses, the doses to radiosensitive off-target organs would be lower than (*i.e.* liver, spleen) or close to (*i.e.* red marrow) the maximum tolerated doses for those organs.

Our work highlights the potential of nanostars as probes for molecular cancer imaging or for the passive delivery of radionuclides for endoradiotherapy. Their high accumulation in tumours with leaky vasculature may aid in selecting patients that will benefit from standard clinical nanomedicine treatments, which currently seem to suffer from low efficacy due to heterogeneity between patients and cancer types. Furthermore, the nanostars may offer opportunities for the palliative treatment of cancer patients, for example when patients appear inoperable due to irresectability of the tumour or when antibody-based therapies are failing due to acquired resistance. As such, this study emphasises the versatility of the nanostars as powerful tools for cancer imaging and therapy.

## Methods

### Materials

Chemicals and anhydrous solvents were purchased from Sigma-Aldrich (Saint Louis, USA), dichloromethane and diethyl ether from Chem-Supply (Gillman, Australia), acryloyl chloride and all other solvents from Merck (Darmstadt, Germany). 2-Aminoethyl-mono-amide-DO3A-tris(*t*-Bu ester) and 1-(4-isothiocyanatophenyl)-3-(6,17-dihydroxy-7,10,18,21-tetraoxo-27-(N-acetylhydroxylamino)-6,11,17,22-tetraazaheptacosane) thiourea (SCN-Bn-DFO) were obtained from Macrocyclics (Plano, USA) and Chelex® 100 resin from Bio-Rad (Hercules, USA). (*E*)-Cyclooct-4-enyl 2,5-dioxo-1-pyrrolidinyl carbonate (TCO-NHS ester) was purchased from Click Chemistry Tools (Scottsdale, USA). 2-Vinyl-4,4-dimethyl-5-oxazolone (VDM) was kindly provided for by Prof. L. Fontaine (University of Maine, Le Mans, France) and 2,2',2''-(10-(2-(tetrazine-poly(ethyleneglycol)_7_-amino)-2-oxoethyl)-1,4,7,10-tetraazacyclododecane-1,4,7,10-tetraacetic acid (Tz-PEG_7_-DOTA) by Dr. S. Poty (MSKCC, New York, USA). Synthesis of RAFT agent 3-(trimethylsilyl)prop-2-yn-1-yl 2-((((3-propionic acid)thio)carbonothioyl)thio)propanoate) (TSPPA) has been described previously 16. [Gd^3+^]2,2',2''-(10-(2-((2-aminoethyl)amino)-2-oxoethyl)-1,4,7,10-tetraazacyclododecane-1,4,7-triyl)triacetic acid (2-aminoethyl-mono-amide-[Gd^3+^]DO3A) was synthesised according to our previously described methods 28. All chemicals were used without further purification, unless otherwise specified. Azobis(isobutyronitrile) (AIBN) was recrystallised from methanol before use.

Polymers were dialysed using Cellu-Sep regenerated cellulose tubular membranes with nominal molecular cut-off weights of 3500 or 12 000-14 000 Da.

Zirconium-89 (^89^Zr) was produced *via* proton beam bombardment of yttrium foil and isolated as high purity [^89^Zr]Zr-oxalate 58. Lutetium-177 (^177^Lu) was purchased from Oak Ridge National Laboratory (Oak Ridge, USA) as [^177^Lu]LuCl_3_ in 0.1M HCl.

### Characterisations

A Grace Reveleris® X2 flash system with silica packed cartridges was used for flash column chromatography.

Instant thin-layer chromatography (iTLC) was performed using silica-impregnated glass microfiber paper (Agilent, Santa Clara, USA) in 0.1M sodium citrate buffer (pH5), unless otherwise stated, and analysed using a BIOSCAN AR 2000.

NMR spectra were measured on a Bruker Avance III 400 MHz (400.13 MHz, 9.4 T) spectrometer at room temperature (Bruker Topspin, v1.3). The residual solvent peak was used as a reference to determine chemical shifts (δ) (7.27 ppm for CDCl_3,_ 4.79 ppm for D_2_O, 2.50 ppm for DMSO-d_6_). Coupling constant (J) and multiplicity (singlet s, doublet d, triplet t, quartet q, multiplet m) were described where possible.

A Shimadzu modular system equipped with a SIL20AD automatic injector, a DGU12A degasser, a CTO10A column oven (40 °C), a LC10AT pump, a RID10A differential refractive-index detector and a SPD10A Shimadzu UV/Vis detector was used for gel permeation chromatography (GPC) Polymer Laboratories, Cirrus v2.0). Samples were measured in *N*,*N*′-dimethylacetamide (HPLC grade, 0.03% w/v LiBr, 1 mL min^-1^), filtered before use (cut-off: 0.45 μm) and ran through a 50 × 7.8 mm guard column (bead size: 5 μm) followed by three KF-805L columns in series (300 × 8 mm linear columns, bead size: 10 μm, pore size maximum: 5000 Å). Linear poly(styrene) standards (0.5-2000 kDa, Polymer Laboratories) were used for calibration.

Dynamic light scattering (DLS) was performed on a Malvern Zetasizer Nano Series equipped with a 4 mW He-Ne laser (λ = 633 nm) and an avalanche photodiode detector (detection angle 173°).

Gadolinium (Gd^3+^) levels (2.0-5.0 mg/ml) were measured by inductively coupled plasma-optical emission spectrometry (ICP-OES) on an OPTIMA 7300 (Western Sydney University, Sydney, Australia) or OPTIMA 7000DV (Hunter College, New York, USA) spectrometer (Perkin Elmer) or by inductively coupled plasma mass spectrometry (ICP-MS) on a Perkin Elmer NexION 300X ICP-MS (University of Missouri, Columbia, USA) using standard operating procedures. Calibration was performed using a five- or six-point calibration curve based on Gd^3+^ standards obtained from Sigma-Aldrich (Saint Louis, USA).

An Agilent 1260 Infinity system equipped with an autosampler (G1367E), binary pump (G1312C), DAD module (G4212A) and an 1100 MSD mass spectrometer, using LC/MSD Chemstation Rev.B.04.03 and Masshunter Easy Access Software were used for ultra-high performance liquid chromatography - mass spectroscopy (LC-MS). For mass spectroscopy, a quadrupole atmospheric pressure ionisation - electrospray (API-ES) source was used with a capillary voltage of 3000 V and a drying gas temperature of 350 °C. For reverse-phase high-performance liquid chromatography (HPLC), a Poroshell 120 EC-C18 column (3.0 × 50 mm, 2.7 μm) was used at 35 °C (injection volume 1 μl; 0.1% formic acid in water, followed by an increasing gradient of 5-100% of 0.1% formic acid in acetonitrile over 3.8 minutes; flow rate 0.500 mL/min).

Radioactive samples were counted using a 2480 Wizard^2^ automatic gamma counter (1 minute per measurement; 800-1000 keV for ^89^Zr, 40-300 keV for ^177^Lu).

### Synthesis and Characterisation of Nanostars

Linear polymer arm **4** (p(BAEA-*co*-OEGA-*co*-VDM)) was synthesised by dissolving *Boc*-protected aminoethyl acrylate (BAEA; **S2**; 5 eq; 1.00 g, 4.65 mmol), poly(ethylene glycol) methyl ether acrylate (OEGA480; **2**; 25 eq; 11.1 g, 23.2 mmol), 2-vinyl-4,4-dimethyl-5-oxazolone (VDM; **3**; 5 eq; 0.647 g, 4.65 mmol), 3-(trimethylsilyl)prop-2-yn-1-yl-2-(((((3-propionic acid)thio)carbonothioyl)thio)propanoate) (TSPPA; **1**; 1 eq; 0.339 g, 0.929 mmol) and 2,2-azobis(2-methylpropionitrile) (AIBN; 0.1 eq; 1.52×10^-2^ g, 9.29×10^-2^ mmol) in 30.2 mL methanol, followed by purging with nitrogen gas at 0°C for 1 hour. The reaction mixture was heated to 70°C and stirred for 7.5 hours under nitrogen atmosphere. The polymerisation was halted by rapidly cooling the reaction mixture to 0°C and exposing it to air. Impurities were removed by three precipitation cycles in 50% diethyl ether in petroleum benzine (BR 40-60°C). Purified product **4** (yellow oil, 7.68 g) was dried using an air flow and analysed by GPC and ^1^H NMR (CDCl_3_).

Cross-linked star polymer **6** (p(BAEA-*co*-OEGA-*co*-VDM)) was formed by stirring a solution of *N*,*N*′-methylenebis(acrylamide) (**5**; 8 eq; 664.8 mg, 4.3 mmol), AIBN (0.33 eq; 29.5 mg, 0.18 mmol) and **4** (1 eq; 5.39×10^3^ mg, 0.54 mmol) in 60 mL toluene under nitrogen atmosphere at 70°C for 24 hours, after purging the reaction mixture with nitrogen gas at 0°C for 1 hour. The reaction was terminated by rapidly cooling the reaction mixture to 0°C and exposing it to air. Impurities were removed by three precipitation cycles in 1/10 (v/v) chloroform/diethyl ether. Purified product **6** (yellow oil, 2.37×10^3^ mg) was dried using an air flow and analysed by ^1^H NMR (CDCl_3_), GPC and DLS.

Gadolinium(III)-functionalised star polymer **7** (p(AEA-*co*-OEGA-*co*-[Gd^3+^]VDMD)) was synthesised by stirring triethylamine (8 arms × 5 VDM × 1.5 eq; 0.283 mL, 2.0 mmol), **S4** (8 arms × 5 VDM × 1.5 eq; 1.22×10^3^ mg, 2.0 mmol) and **6** (1 eq; 2.37×10^3^ mg, 0.034 mmol) in 5.8 mL DMF for 48 hours. After removing all solvent and triethylamine *in vacuo*, a large excess of 85% phosphoric acid solution was added and the reaction mixture was stirred for 30 minutes. Purified product **7** (1.42×10^3^ mg**)** was obtained after dialysis in water and was stored in solution at 4°C. The product was characterised by ^1^H NMR (CDCl_3_), DLS and ICP-OES or ICP-MS.

### Radiolabeling of Nanostars with ^89^Zr

Star polymers were functionalised with deferoxamine (DFO) by mixing p(AEA-*co*-OEGA-*co*-[Gd^3+^]VDMD) (**7**; 1 eq; 55 mg, 6.5×10^-4^ mmol) in 5 mL 0.1M NaHCO_3_ (pH 9) with SCN-Bn-DFO (10 eq; 5 mg, 6.5×10^-3^ mmol) in 50 µL DMSO at 40°C for 16 hours. Small molecular weight impurities were removed by dialysis in water and large molecular weight impurities using PTFE syringe filters (0.1 µm, GE Healthcare). Purified p(DFO-AEA-*co*-OEGA-*co*-[Gd^3+^]VDMD) star polymer (53.1 mg) was stored in solution at 4°C until further use and analysed by ^1^H NMR (DMSO-d_6_) and DLS.

[^89^Zr]Zr-oxalate (~90 MBq) was neutralised using 1M Na_2_CO_3_ and subsequently mixed with p(DFO-AEA-*co*-OEGA-*co*-[Gd^3+^]VDMD) star polymer (120 μg) in 360 μL 0.1M HEPES buffer (pH 7.4). Desired product p([^89^Zr]Zr-DFO-AEA-*co*-OEGA-*co*-[Gd^3+^]VDMD) star polymer (**8**) was obtained after 30 minutes incubation at 37°C and used without further purification. Radiochemical purity was measured by iTLC. *In vitro* stability of star polymer **8** (p([^89^Zr]Zr-DFO-AEA-*co*-OEGA-*co*-[Gd^3+^]VDMD)) with respect to radiochemical purity and demetallation was assessed after overnight incubation of the nanostar in saline, PBS and human AB-type serum at 37°C (*n* = 3).

### Radiolabeling of Nanostars with ^177^Lu

Star polymers were functionalised with *trans*-cyclooctene (TCO) by stirring p(AEA-*co*-OEGA-*co*-[Gd^3+^]VDMD) (**7**, 1 eq; 95 mg, 1.1×10^-3^ mmol) in the presence of TCO-NHS ester (8 arms × 4 AEA × 1.2 eq; 11.6 mg, 4.4×10^-2^ mmol) in 27 mL DMF at 60°C for 16 hours. Small molecular weight impurities were removed by dialysis in 5% DMSO and large molecular weight impurities using PTFE syringe filters (0.1 µm, GE Healthcare). Purified TCO-functionalised star polymer **9** (p(TCO-AEA-*co*-OEGA-*co*-[Gd^3+^]VDMD)) was stored in solution at 4°C and analysed by ^1^H NMR (D_2_O) and DLS.

Tz-PEG_7_-DOTA (**10**; 1 eq; 15 μg, 12.5 nmol) was added to [^177^Lu]LuCl_3_ (~95 MBq) in 100 μL 0.25M NH_4_OAc buffer in chelex water (pH 5.5) and mixed for 60 minutes at 37°C. TCO-functionalised star polymer **9** (8 arms × 4 TCO × 1.5 eq; 52.6 μg, 0.6 nmol) in 45 μL was added and the mixture was allowed to react for 30 minutes at 37°C. Purified radiolabelled star polymer **11** (p([^177^Lu]Lu-DPAEA-*co*-OEGA-*co*-[Gd^3+^]VDMD)) was obtained by centrifugal filtration (Amicon Ultra-4, 50k) using saline. Radiochemical purity was measured by iTLC using 50% ethanol as mobile phase.

### Cell Culture

CT26 colon cancer cells were cultured in Roswell Park Memorial Institute (RPMI)-1640 growth medium, supplemented with 10% fetal calf serum (FCS). BxPC3 pancreatic cancer cells were cultured in RPMI-1640 growth medium, supplemented with 10% FCS and 2 mM L-glutamine. Both cell culture media were supplemented with 100 units mL^-1^ penicillin and streptomycin.

### Tumour Models

All animals were treated according to the guidelines approved by the Research Animal Resource Center and Institutional Animal Care and Use Committee at Memorial Sloan Kettering Cancer Center. Six-to-eight week old female BALB/c or athymic nude mice (Charles River Laboratories, New York, USA) were injected subcutaneously with CT26 cells (1 × 10^5^ cells) or BxPC3 cells (5 × 10^6^ cells) in 150 µL 1:1 growth media/Matrigel^®^ (BD Biosciences, San Jose, CA), respectively. Cells were injected using a sterile syringe equipped with a 28-gauge needle in either right flank. Mice were allowed to acclimatise to laboratory conditions for 1 week prior to injection of the tumour cells and were housed in type II polycarbonate cages at 22 °C (60% relative humidity) in a 12 h light - 12 h dark cycle, while providing a sterilised standard laboratory diet and sterile water *ad libitum*. Positron emission tomography (PET) imaging and biodistribution studies were performed within 2-5 weeks after injection, when tumour volumes reached 100-250 mm^3^. Tumour volume *V* was estimated by Vernier calliper measurements of the longest tumour axis *a* and its perpendicular axis *b*, using the formula *V* = (4*π*/3) × (*a*/2)^2^ × (*b*/2).

At defined time points prior to imaging or *ex vivo* biodistribution studies, mice were injected with 15 µg p([^89^Zr]Zr-DFO-AEA-*co*-OEGA-*co*-[Gd^3+^]VDMD) star polymer **8** (10 MBq, MA = 64 GBq/µmol), or 15 µg p([^177^Lu]Lu-DPAEA-*co*-OEGA-*co*-[Gd^3+^]VDMD) star polymer **11** (0.8 MBq, MA = 5 GBq/µmol) in 130-150 µL saline (0.9% NaCl). All compounds were administered by tail vein injection.

### ^1^H Relaxivity and Magnetic Resonance Imaging (MRI)

A ^1^H nuclear magnetic resonance dispersion (NMRD) profile was obtained at 310 K over a continuum of magnetic fields from 0.24 mT to 0.94 T (0.01 - 40 MHz) using a Spinmaster FFC-2000 (fast field cycling) NMR relaxometer (Stelar, Mede PV, Italy). An aqueous suspension of the Gd^3+^-labelled nanostars (1 mL) was placed in a 10 mm NMR tube and equilibrated at the set temperature for 15 minutes prior to conducting any experiments. The recycle delay was set to ≥ 5 *T*_1_, and the signal was averaged over four scans for all measurements. Relaxivities were calculated by subtracting the diamagnetic contribution to the relaxation rates (*i.e.* relaxation rates of water without the paramagnetic contrast agent) from the measured relaxation rates, and normalising the values to the Gd^3+^ concentration. The nonlinear least-squares fitting of the ^1^H NMRD data was performed using the Levenberg-Marquardt algorithm implemented in Origin 2018 software (Originlab Corporation, Massachusetts, USA).

For 3 T *T*_1_-weighted MRI imaging and relaxivity measurements, a dilution series of Gd^3+^-labelled nanostars in HEPES buffer in 5 mm NMR tubes (500 µL) or anaesthetised mice (*n* = 3; oxygen gas mixture with 1.5-2% isofluorane (Baxter Healthcare, Deerfield, USA)), injected with 3 mg or 10 mg of nanostar **6** (p(BAEA-*co*-OEGA-*co*-VDM)), were positioned in a ^1^H transmit/receive volume coil with quadrature detection at the isocentre of a 3 T BioSpec MRI scanner (Bruker). For the phantom studies, *T_1_*-weighted MRI images were acquired using a gradient echo sequence (*T_R_*= 22.6 ms, *T_E_*= 3.6 ms, 30 mm × 10 mm field of view (96 × 32 resolution), 2 mm slice thickness, 10 averages) at a 21-degree pulse angle. For the *in vivo* studies, *T_1_*-weighted MRI images were acquired using a gradient echo sequence (*T_R_*= 21.955 ms, *T_E_*= 3.003 ms, 30 mm × 30 mm field of view (96 × 96 resolution), 2 mm slice thickness, 30 averages) at a 15-degree pulse angle. Relaxivity of the nanostars at 3 T was calculated from water proton *T*_1_ measurements at varying nanostar concentrations (0 mg/mL, 0.125 mg/mL, 0.25 mg/mL, 0.5 mg/mL, 1 mg/mL). *T*_1_ values of these solutions were acquired with a Look-Locker *T*_1_-map sequence across an axial 2 mm slice with a 20 mm × 20 mm field of view (160 × 160 resolution), 10 ms echo time, 4000 ms dummy duration, 1 average and repetition times of 2000, 1500, 1000, 700, 500, 300, 150 and 50 ms. *T*_1_-maps were generated in Paravision 6.0.1 (Bruker), regions of interest (ROI) were drawn around the areas corresponding to each individual sample and ROIs were integrated to calculate *T*_1_. These *T*_1_ values were subsequently used to calculate relaxivity (*r*_1_). For *in vivo* imaging studies, MR image volumes were co-registered with the subsequent PET/CT scans of each mouse using Elastix deformable image registration within open source 3D Slicer software (v4.8.1). *T*_1_-weighted contrast 3 days after injection of the nanostars was compared to baseline pre-injection MR images.

Relaxivity of the Gd^3+^-labelled nanostars (5.95×10^-8^ mol Gd^3+^) at 7 T was compared with the relaxivity of Gadovist in 5 mm NMR tubes (550 µL), positioned in a 40 mm two channel ^1^H/^19^F volume coil at the isocentre of a simultaneous PET/MRI system (Bruker 7 T Clinscan interfaced with a Siemens Spectrometer running Numaris/4 VB17 with a PET ring positioned at the isocenter of the magnet consisting of three rings of 16 crystal blocks), while acquiring *T*_1_-weighted images using a 3D VIBE sequence (*T_R_*=12ms, *T_E_*=0.93ms, in-plane resolution=0.312mm) at 5 different flip angles.

### Positron Emission Tomography (PET) Imaging

Mice (*n* = 4-5 per timepoint) were anesthetised 5-10 minutes prior to scanning *via* inhalation of a 1.5-2% isofluorane in oxygen gas mixture. PET images were acquired on an Inveon small-animal micro-PET/CT scanner (Siemens Medical Solutions, Knoxville, USA) or a Focus 120 small-animal PET scanner (Siemens Medical Solutions, Knoxville, USA) under 1.5-2% isofluorane/oxygen anaesthesia using a dedicated quadruple animal scanning platform for simultaneous scanning. PET data were recorded using a minimum of 15 million coincident events (~ 15 minutes) with an energy window of 350-650 keV and a coincidence-timing window of 6 ns. Data were sorted into 2-dimensional histograms by Fourier rebinning, and transverse images were reconstructed by filtered back-projection or 2-dimensional ordered subset expectation maximisation (2D-OSEM) into a 128 x 128 matrix. The image data were normalised to correct for nonuniformity of response of the PET, dead-time count losses, positron branching ratio, and physical decay to the time of injection, but no attenuation, scatter, or partial-volume averaging correction was applied. Individual image volumes were constructed using the cropping function of the Inveon Research Workspace software (Siemens Medical Solutions, Knoxville, USA). Activity concentrations (%ID/g) were obtained by conversion of the counting rates in the reconstructed images using a system calibration factor, derived from a mouse-sized water-equivalent phantom containing ^89^Zr. Further image processing was performed using 3D Slicer software (v4.8.1). Intensity scales for both the high EPR (CT26) and low EPR tumour (BxPC3) models were adjusted to an apparently similar liver and spleen uptake for both models, warranting the fair comparison of PET data obtained from mice with two different backgrounds (*i.e.* BALB/c and athymic nude mice). Three-dimensional volumes of interest (VOIs) were defined through semi-automatic segmentation techniques and activity concentrations were quantified by selecting the maximal intensity voxel per VOI.

### *Ex Vivo* Biodistribution

Mice (*n* = 4-5 per timepoint) were euthanised *via* CO_2_ asphyxiation and organs of interest (including tumour tissue) were removed, rinsed in water and dried in air for several minutes, before tissues were weighed and counted using a gamma counter. Measured counts were converted into radioactivity units using a calibration curve obtained from known standards for ^89^Zr or ^177^Lu. The injected dose per tissue mass (%ID/g) was calculated after background- and decay-correction to the time of radioligand injection.

### Dosimetry and Therapy Studies

Dosimetry studies were performed according to procedures described previously 51. Briefly, the time-integrated activity coefficients (*i.e.* residence times) per organ were calculated by trapezoidal integration of the time-activity curves for each organ, based on biodistribution data for the p([^177^Lu]Lu-DPAEA-*co*-OEGA-*co*-[Gd^3+^]VDMD) star polymer (**11**). Clearance of activity following the last measured time point was assumed to be due to radioactive decay only. Resulting organ time-integrated activity coefficients were entered into OLINDA software (v2.0) to estimate mean organ-level absorbed doses (Gy/MBq) for the ICRP89 adult male model. These methods assume that the standardised uptake values are invariant between organs in mice and humans 56.

Tissue absorbed dose estimates for mice assume self-absorbed fractions of 1 for weakly-penetrating raditions and 0 for photons. Optimal mouse therapy doses were estimated from maximum tolerated doses for all organs. BALB/c mice with CT26 colon cancer isografts were randomized in five cohorts (*n* = 8 per cohort) with approximately equal average tumour volumes. To minimize bias due to outliers, the two mice with the smallest and largest tumours were excluded from each therapy cohort (Figure S17). One day after randomization, mice were injected with 1.5 MBq, 3.7 MBq or 7.4 MBq of p([^177^Lu]Lu-DPAEA-*co*-OEGA-*co*-[Gd^3+^]VDMD) star polymer (**11**; 23 µg per mouse). Mice in the two control groups were injected either with vehicle (0.9% sterile saline) or non-radioactive nanostar **6** (p(BAEA-*co*-OEGA-*co*-VDM); 23 µg per mouse). Tumour volumes and body weights were measured twice per week until tumour volume *V* > 2000 mm^3^.

### Toxicity studies

Mice were monitored for outward signs of toxicity, including lethargy and loss of appetite. Blood samples (50-100 µL, n = 3 per cohort) were collected retroorbitally twice per week and analysed using an Hemavet 950 (Drew Scientific). Potential haematological toxicity was assessed by comparing red blood cell counts, haematocrit values, platelet counts and white blood cell counts between cohorts and with baseline measurements one day prior to injection.

### Statistical Analysis

Data were compared using a two-tailed independent-samples t-test, for which equal variances were not assumed if Levene's Test for Equality of Variances was significant. Cumulative survival was defined as the time in days after injection until euthanisation. Statistical tests and survival analyses were performed using SPSS Statistics software (v25, IBM, Armonk, USA) or RStudio Statistics 3.6.0 software (RStudio Inc., Boston, USA) and were considered significant at the 95% confidence interval (*P*<0.05).

## Figures and Tables

**Table 1:** Molecular parameters of the nanostars obtained from the theoretical fitting of the NMRD data at 37 °C.

Parameter	Nanostar
*r*_1*,max*_ (mM^-1^s^-1^)	24.4
*τ*_M_ (ns)	853 ± 43
*τ*_R_ (ns)	3.9 ± 1.4
*τ*_so_ (ps)	196 ± 11
*τ*_v_ (ps)	42 ± 2
*q*_ss_	2.8 ± 0.2

*r*_1,max_: maximal relaxivity, *τ_M_*: water exchange rate, *τ_R_*: reorientational correlation time, *τ*_so_: electronic relaxation time at zero field, *τ*_v_: electronic relaxation correlation time, *q*_ss_: number of water molecules in second coordination sphere.

**Table 2:** Murine organ-level absorbed dose coefficients and therapeutic indices calculated from the biodistribution data of the ^177^Lu-labelled nanostars.

Organ	Absorbed dose (Gy/MBq)	Therapeutic index	
Tumour	1.94	-	
Blood	1.63	1.19	
Brain	0.04	41.23	
Lungs	0.85	2.27	
Liver	6.87	0.28	
Spleen	4.55	0.43	
Pancreas	0.32	6.09	
Stomach	0.22	8.86	
Intestines	0.14	13.89	
Kidneys	0.78	2.47	
Bone	0.34	5.72	

## Abbreviations

AIBN: azobis(isobutyronitrile)
API-ES: atmospheric pressure ionisation - electrospray
BAEA: *Boc*-protected aminoethyl acrylate
DFO: desferoxamine
DLS: dynamic light scattering
DO3A: 1,4,7,10-tetraazacyclododecane-1,4,7-triyl)triacetic acid
DOTA: 1,4,7,10-tetraazacyclododecane-1,4,7,10-tetraacetic acid
DPAEA: DOTA-PEG_7_-aminoethyl acrylate
EPR: enhanced permeability and retention
FCS: fetal calf serum
GPC: gel permeation chromatography
HPLC: high-performance liquid chromatography
ICP: inductively coupled plasma
ICP-MS: inductively coupled plasma mass spectrometry
ICP-OES: inductively coupled plasma - optical emission spectrometry
iTLC: instant thin layer chromatography
LC-MS: liquid chromatography - mass spectroscopy
MA: molar activity
MRI: magnetic resonance imaging
MW: molecular weight
NHS: *N*-hydroxysuccinimide
NMRD: nuclear magnetic resonance dispersion
OEGA: oligoethylene glycol methyl ether acrylate
PDI: polydispersity index
PEG: poly(ethylene glycol)
PET: positron emission tomography
RAFT: reversible addition-fragmentation chain-transfer
ROI: region of interest
SCN: thiocyanate
TCO: *trans*-cyclooctene
TSPPA: 3-(trimethylsilyl)prop-2-yn-1-yl 2-((((3-propionicacid)thio)carbonothioyl)thio)propanoate)
Tz: tetrazine
VDM: 2-vinyl-4,4-dimethyl-5-oxazolone
VDMD: VDM-DOTA
VOI: volume of interest
