# Supplementary material for: Delivery of polymeric nanostars for molecular imaging and endoradiotherapy through the enhanced permeability and retention (EPR) effect
Source: Theranostics. 2020 Jan 1;10(2):567–84. doi: 10.7150/thno.36777 (PMC6929988; doi:10.7150/thno.36777)
Supplement: Supplementary file 1 — Supplementary figures and tables. [file thnov10p0567s1.pdf]

# - Supporting Information -

Delivery of polymeric nanostars for molecular imaging and endoradiotherapy through the enhanced permeability and retention (EPR) effect

Jeroen A.C.M. Goos[1,2], Andrew Cho[3,4], Lukas M. Carter[1], Thomas R. Dilling[1], Maria Davydova[1], Komal Mandleywala[1], Simon Puttick[5], Abhishek Gupta[6], William S. Price[6], John F. Quinn[2], Michael R. Whittaker[2], Jason S. Lewis[1,7,8,*], Thomas P. Davis[2,9,*]

[1]*Department of Radiology, Memorial Sloan-Kettering Cancer Center, New York, USA,* [2]*ARC Centre of Excellence in Convergent Bio-Nano Science & Technology, Monash Institute of Pharmaceutical Sciences, Monash University, Parkville, Australia,* [3]*Department of Biochemistry & Structural Biology, Weill Cornell Graduate School, New York, USA,* [4]*Weill Cornell/Rockefeller/Sloan Kettering Tri-Institutional MD-PhD Program, New York, USA,* [5]*Probing Biosystems Future Science Platform, Commonwealth Scientific and Industrial Research Organisation, Herston, Australia,* [6]*Nanoscale Organisation and Dynamics Group, Western Sydney University, Penrith, Australia,* [7]*Department of Radiology, the Molecular Pharmacology Program and the Radiochemistry and Molecular Imaging Probes Core, Memorial Sloan Kettering Cancer Center, New York, USA,* [8]*Departments of Radiology and Pharmacology, Weill Cornell Medical College, New York, USA,* [9]*Australian Institute for Bioengineering and Nanotechnology, University of Queensland, St Lucia, Australia.*

* Corresponding authors

Synthesis of *tert*-butyl (2-hydroxyethyl)carbamate (S1)

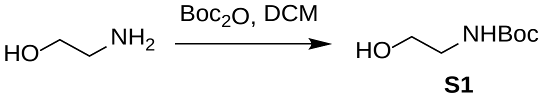

A solution of 2-aminoethanol (2.39 mL, 39.6 mmol) in 50 mL anhydrous dichloromethane was added drop-wise to a solution of di-*tert*-butyl dicarbonate (10 mL, 43.5 mmol) in 10 mL anhydrous dichloromethane under nitrogen atmosphere. The reaction mixture was stirred for 4.5 hours at room temperature, then washed with a saturated aqueous solution of $NaHCO_3$, dried over $MgSO_4$, filtered and concentrated *in vacuo*. The crude product was purified by flash column chromatography (gradient 10/90 – 50/50 v/v ethyl acetate/petroleum spirit (BR 40-60°C)) to afford **S2** (4.24 g, 66% yield). ^{1}H NMR (400 MHz, $CDCl_3$), δ (ppm): 3.68 (t, J=3.68 Hz, 2H), 3.27 (t, J=3.27 Hz, 2H), 1.44 (s, 9H).

Synthesis of 2-((*tert*-butoxycarbonyl)amino)ethyl acrylate (S2)

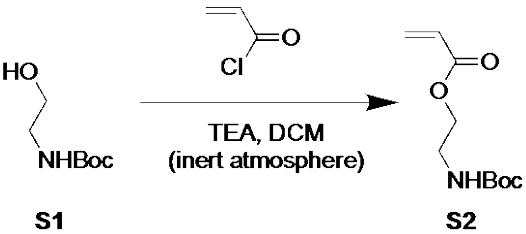

Acryloyl chloride (1.78 mL, 21.9 mmol), triethyl amine (4.58 mL, 32.9 mmol) and **S1** (4.24 mg, 26.3 mmol) were dissolved in 130 mL anhydrous dichloromethane. Under nitrogen atmosphere, the reaction mixture was stirred at room temperature for 1 hour. Pure product **S2** was obtained by flash column chromatography (gradient 5/95 – 25/75 v/v ethyl acetate/petroleum benzine (BR 40-60°C)) as a white solid (4.38 g, 93% yield). LC-MS calculated 215.1, found 237.9 (M + Na^+). ^{1}H NMR (400 MHz, $CDCl_3$), δ (ppm): 6.43 (dd, J_1=1.2, J_2=17.2, 1H), 6.13 (dd, J_1=10.4, J_2=17.2,

1H), 5.86 (dd, J_1=1.6, J_2=10.4, 1H), 4.22 (t, J=5.2, 2H), 3.43 (q, J_1=J_2=5.2, 1H), 1.44 (s, 9H). ^{13}C (101 MHz, $CDCL_3$) δ (ppm): 166.16 ($C=O_{acryate}$), 155.88 ($C=O_{Boc}$), 131.33 ($CH_{2,acrylate}$), 128.20 ($CH_{acrylate}$), 79.72 (Cq), 63.89 (CH_2), 39.80 (CH_2), 28.51 (CH_3).

Synthesis of [Gd^{3+}]2,2',2''-(10-(2-((2-aminoethyl)amino)-2-oxoethyl)-1,4,7,10-tetraazacyclo-dodecane-1,4,7-triyl)triacetic acid (S4, 2-aminoethyl-mono-amide-[Gd^{3+}]DO3A)

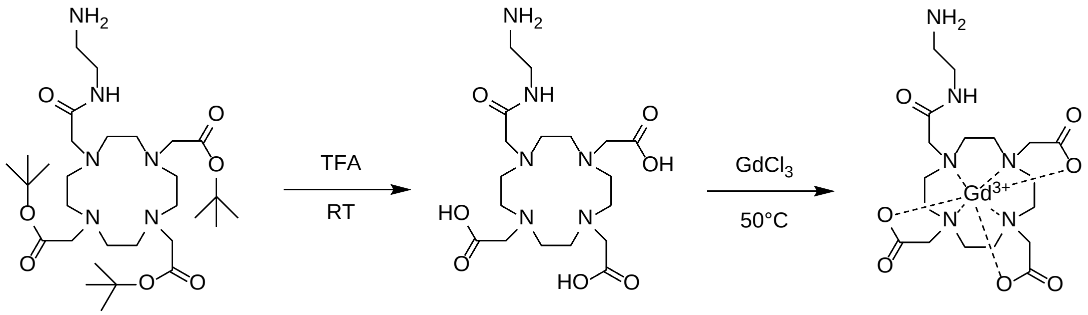

S3 **S4**

1-(5-amino-3-aza-2-oxypentyl)-4,7,10-tris(*tert*-butoxycarbonylmethyl)-1,4,7,10-tetraazacyclododecane (DO3A-*t*Bu-NH_2) (415.3 mg, 0.60 mmol) was stirred in 10 mL trifluoroacetic acid for 16 hours. After removing all trifluoroacetic acid *in vacuo*, the intermediate product (**S3**, yellowish oil) was dissolved in 15 mL water. The pH was adjusted to 6 using 2N KOH. Gadolinium(III)chloride (236.0 mg, 0.90 mmol) was added and the reaction mixture was stirred for 2 days at 50°C, while maintaining the pH at 6 using 2N KOH. The excess of gadolinium(III)chloride was removed by stirring the reaction mixture with a large excess of Chelex® 100 resin for several hours, followed by filtration with water. The final product was obtained after lyophilisation of the filtrate (355.8 mg, 99%). LC-MS calculated 601.1, found 601.9 (M + H^+). ^1H NMR of intermediate product (400 MHz, $CDCl_3$), δ (ppm): 8.72 (s, 1H), 7.91 (s, 3H), 4.07 (s, 2H), 3.90 (s, 2H), 3.80-3.49 (m, 4H), 3.49-3.27 (m, 10H), 3.27-2.98 (m, 8H), 2.93 (q, J_1=J_2=5.6, 2H), 1.53 (s, 2H).

Synthesis of fluorescent star polymer (S6)

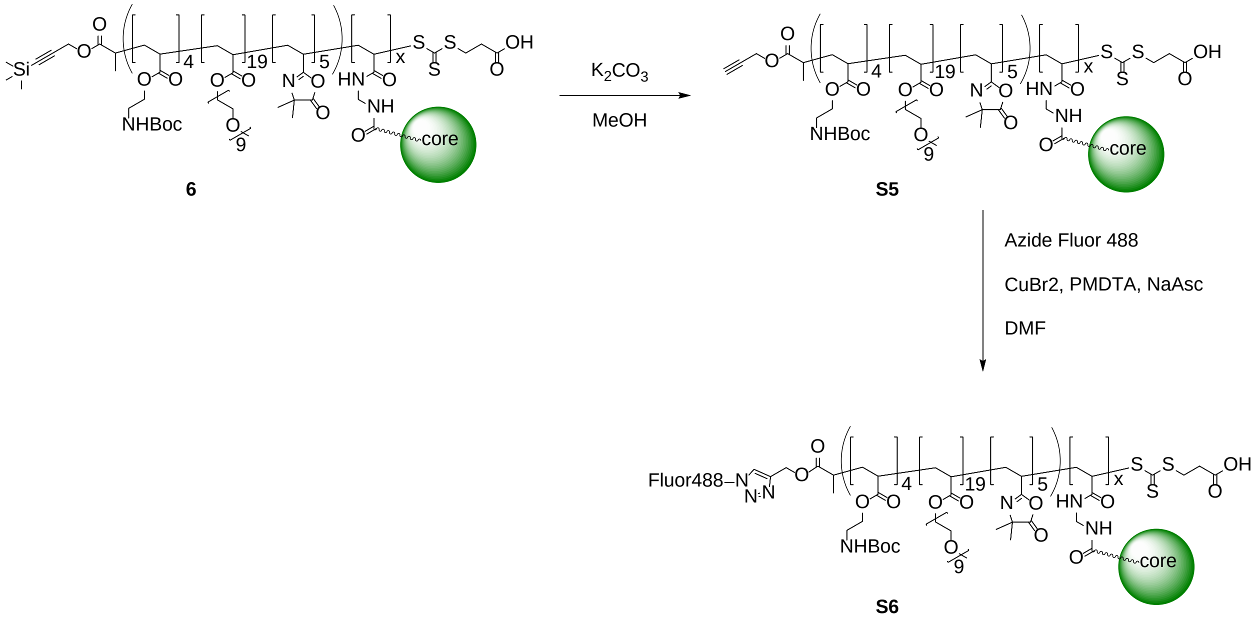

Star polymer **6** (p(BAEA-*co*-OEGA-*co*-VDM); 1 eq; 100 mg, 1.2×10^{-3} mmol) was dissolved in 2 mL methanol and stirred under nitrogen atmosphere in the presence of K_2CO_3 (8 arms × 0.5 eq; > 0.66 mg, 4.8×10^{-2}) for 4 hours at room temperature. 1% Acetic acid in methanol was used to neutralise the reaction mixture, after which all solvents were removed *in vacuo*. The intermediate product **S5** was analyzed by ^{1}H NMR ($CDCl_3$) and GPC (Fig. S3).

In a reaction vial protected from light, copper bromide (>0.03 mg, 1.4×10^{-4} mmol), *N*,*N*,*N′*,*N″*,*N″*-pentamethyldiethylenetriamine (>0.02 mg, 1.4×10^{-5} mmol), sodium ascorbate (E301; >0.05 mg, 4.3×10^{-4} mmol), **S5** (20.0 mg, 2.4×10^{-4} mmol) and azide Fluor 488 (1.1 mg, 1.9×10^{-3} mmol) were dissolved in 0.2 mL DMF. The reaction mixture was purged with nitrogen gas for 30 minutes and stirred for at least 24 hours under nitrogen atmosphere at room temperature. The click reaction between Fluor 488 and p(BAEA-*co*-OEGA-*co*-VDM) star polymer (**6**) was confirmed by comparing GPC traces at 254 nm and 488 nm (Fig. S3).

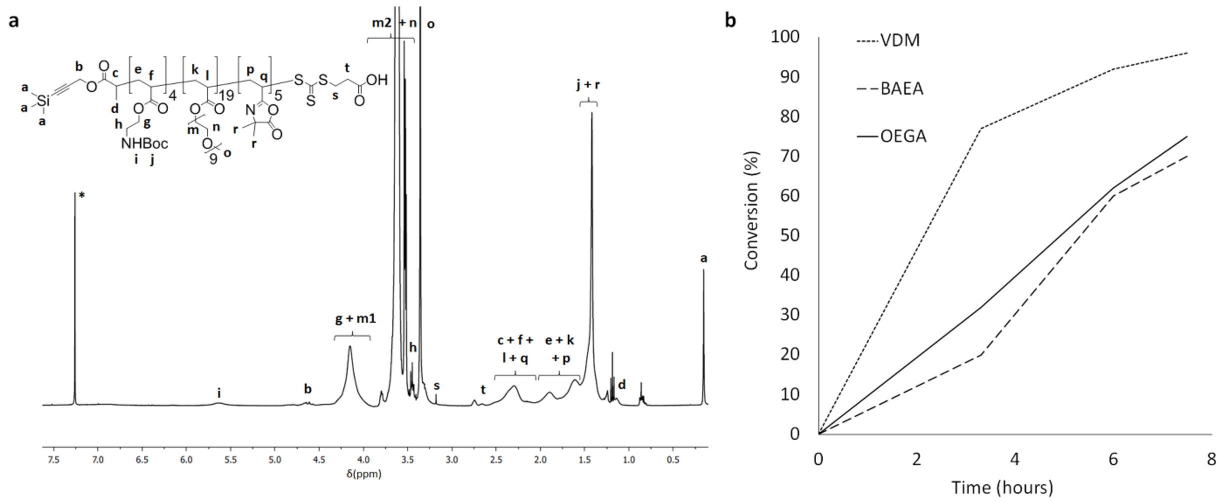

Figure S1. Characterisation of p(BAEA-*co*-OEGA-*co*-VDM) arm polymer **4** by ^{1}H NMR. (a) ^{1}H NMR spectrum of purified arm polymer **4** in $CDCl_3$ (residual solvent peak indicated by *). (b) Polymerisation kinetics calculated from the crude ^{1}H NMR spectrum. VMD monomer units were incorporated in the arms faster than OEGA and BAEA monomers, creating a gradient of VDM monomer units from one end of the polymer arm.

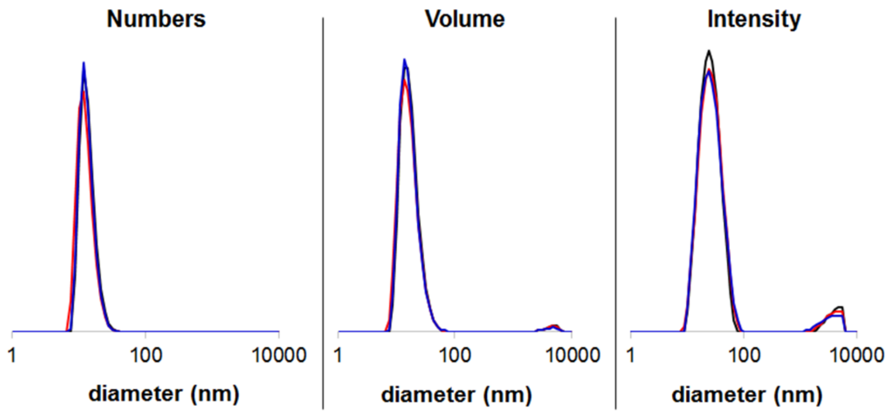

Figure S2. Size distribution profile of p(BAEA-*co*-OEGA-*co*-VDM) star polymer (**6**; D_h = 13 nm), as determined by dynamic light scattering (DLS) (n=3). D_h: Number-average hydrodynamic diameter.

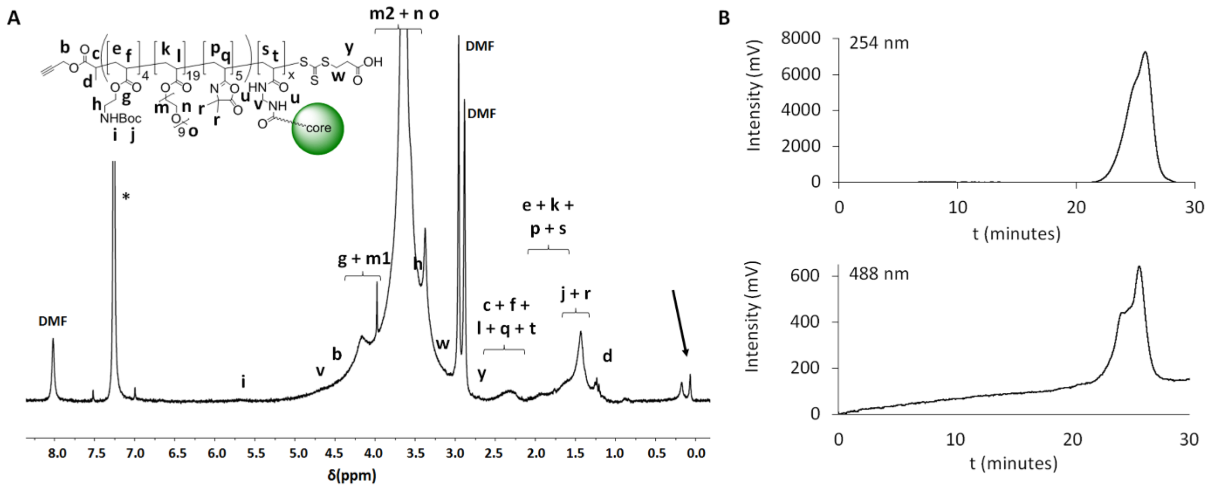

Figure S3. (A) ^{1}H NMR spectrum of alkyne-deprotected p(BAEA-*co*-OEGA-*co*-VDM) star polymer (**S5**) in $CDCl_3$ (traces of DMF are still visible, residual solvent peak indicated by *). Significant reduction of the peak at δ = 0.06 ppm indicates deprotection of the alkyne end group (arrow). (B) Gel permeation chromatogram (GPC) of p(BAEA-*co*-OEGA-*co*-VDM) star polymer functionalised with fluorescent dye *Fluor 488* (**S6**). The UV trace at 488 nm (bottom panel) confirms that the nanostars (elution at 22-27 min) have been fluorescently labelled. The asymmetric peak indicates that some aggregation has occurred.

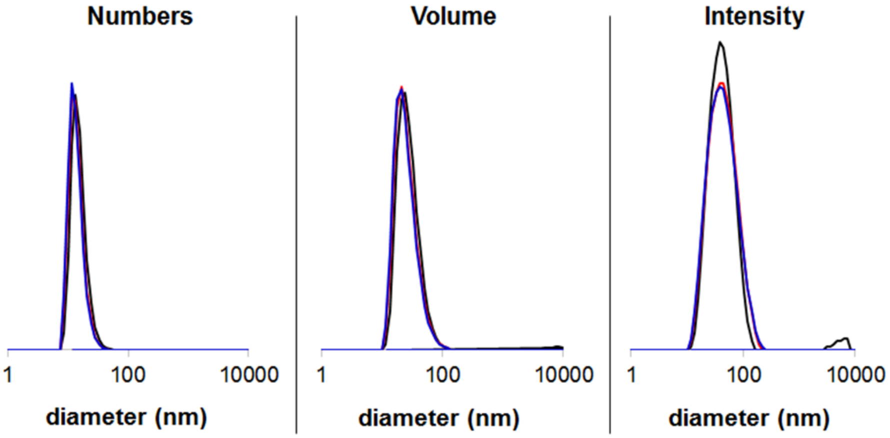

Figure S4. Size distribution profiles of p(AEA-*co*-OEGA-*co*-[Gd^{3+}]VDMD) star polymer (**7**; D_h = 11 nm), as determined by dynamic light scattering (DLS) (n=3). D_h: Number-average hydrodynamic diameter.

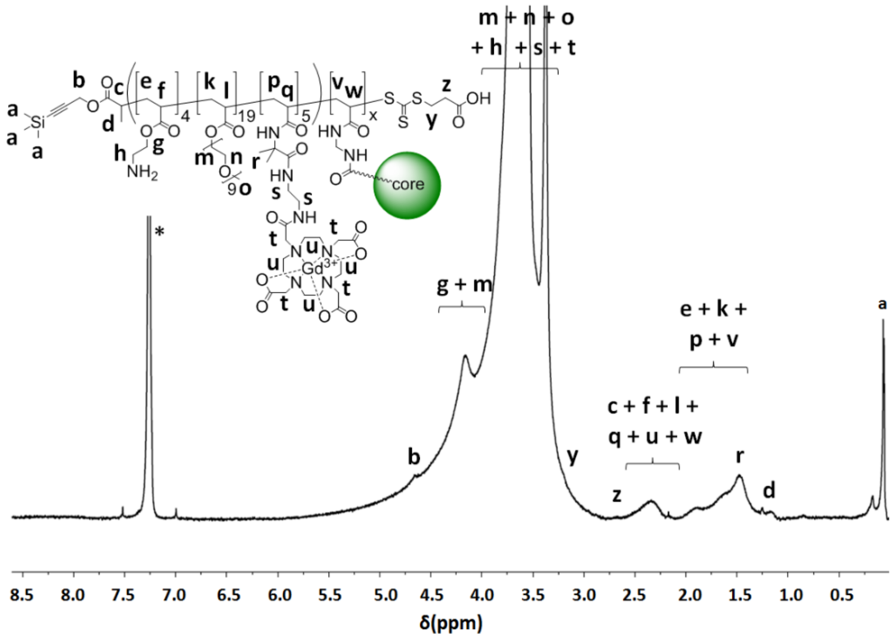

Figure S5. ^{1}H NMR spectrum of p(AEA-*co*-OEGA-*co*-[Gd^{3+}]VDMD) star (**7**) in $CDCl_3$ (residual solvent peak indicated by *). The presence of Gd^{3+} causes significant peak broadening.

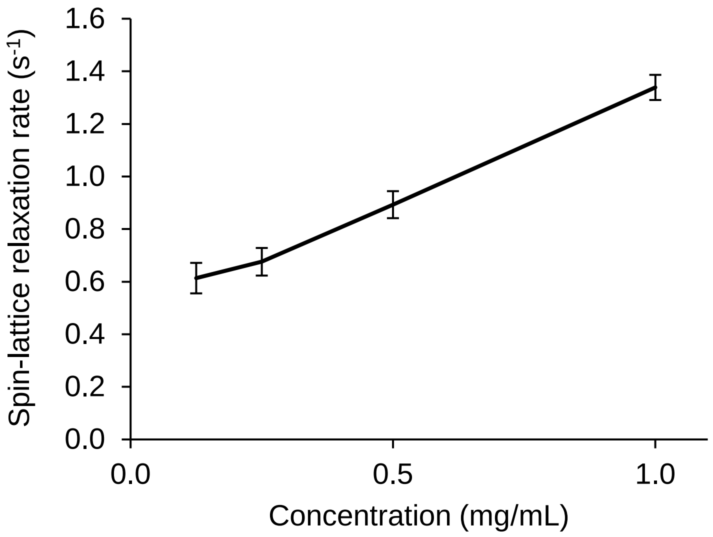

Figure S6. Spin-lattice relaxation rate of water protons in the presence of increasing concentrations of nanostar **7** (p(AEA-*co*-OEGA-*co*-[Gd^{3+}]VDMD). Spin-lattice relaxation rates were obtained from an increasing dilution series of **7** in aqueous buffer at 3 T.

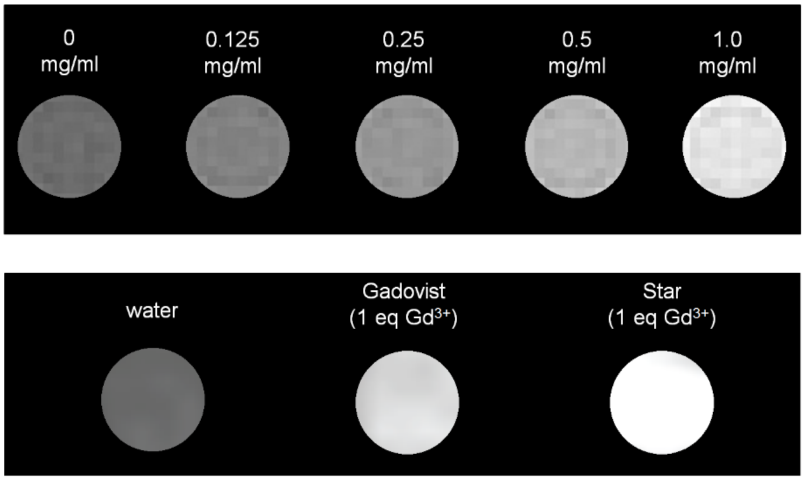

Figure S7. A dilution series of the star polymers in water at 3 T showed an increasing T_1-weighted contrast with increasing concentrations. At equimolar Gd^{3+} concentrations, the nanostars demonstrated enhanced T_1-weighted contrast at 7 T compared to water (4.2 times shorter T_1 for nanostars) and Gadovist (1.7 times shorter T_1 for nanostars).

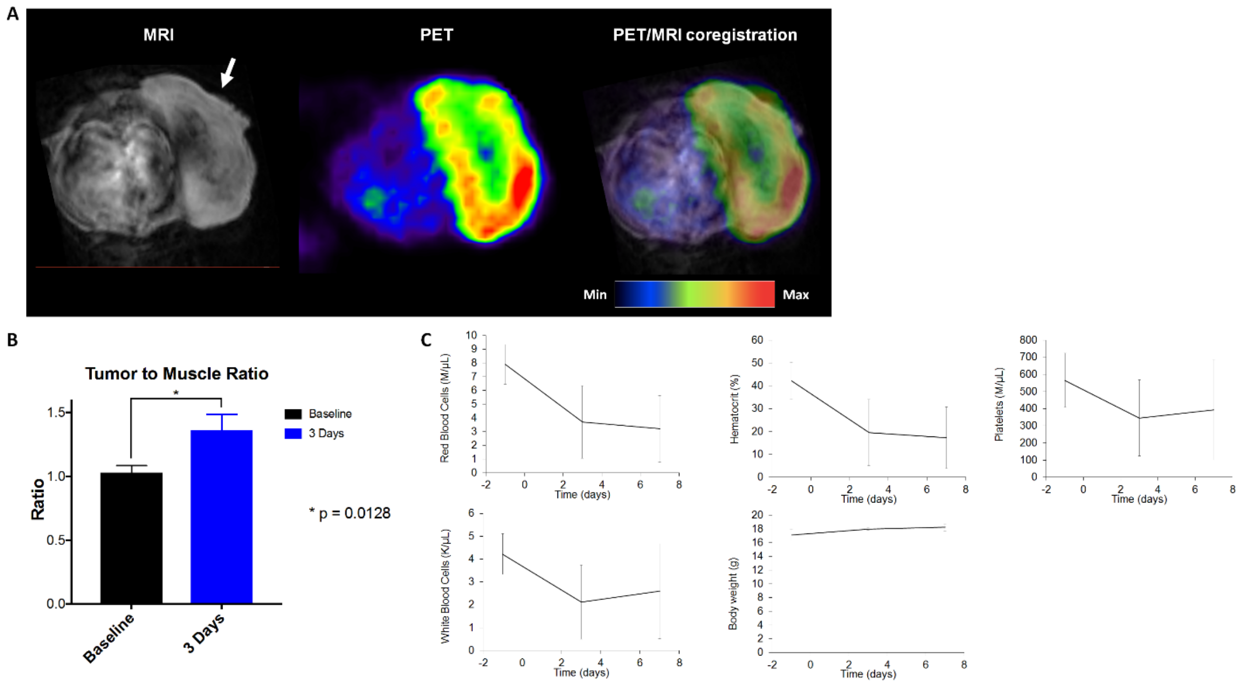

Figure S8. (A) Enhanced T_1-weighted contrast was observed in BALB/c mice (n=3) carrying CT26 tumours, 3 days after the injection of nanostar **6** (p(BAEA-*co*-OEGA-*co*-VDM)) (3 mg; tumour indicated by white arrow). PET images were obtained by co-injection of ^{89}Zr-labelled nanostar **8** (p([^{89}Zr]Zr-DFO-AEA-*co*-OEGA-*co*-[Gd^{3+}]VDMD)) (B) Contrast enhancement in the tumour was significantly higher 3 days after the injection of the nanostars compared to contrast in the pre-injection (baseline) images (P=0.013). (C) The haematological toxicity of the nanostars was assessed by measuring alterations in red blood cell counts, haematocrit values, platelet counts and white blood cell counts. Further, systemic toxicity was monitored by measuring signs of lethargy, loss of appetite and body weight.

A

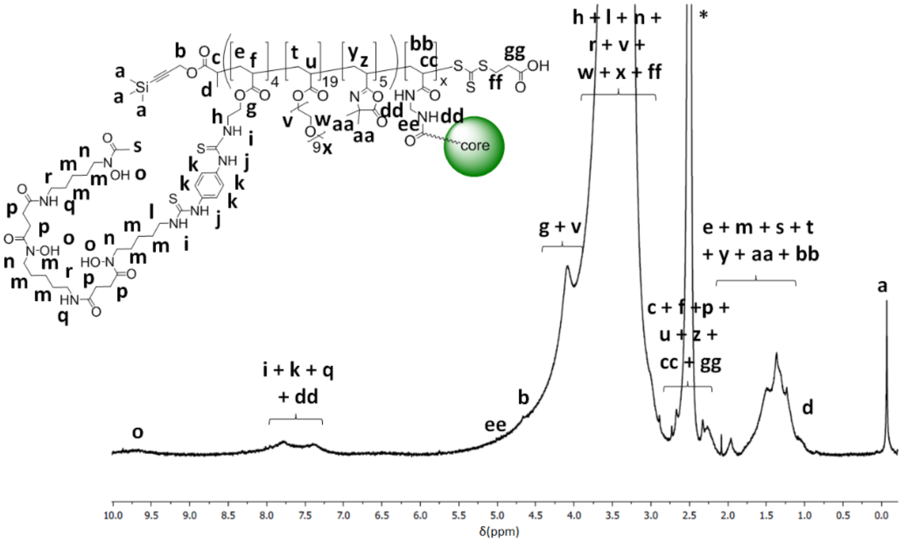

B

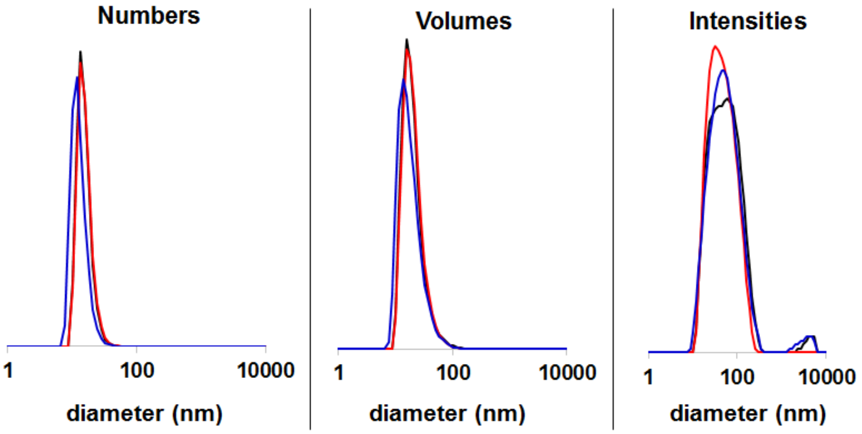

Figure S9. (A) ^{1}H NMR spectrum of p(DFO-AEA-*co*-OEGA-*co*-[Gd^{3+}]VDMD) star polymer in DMSO-d_6 (residual solvent peak indicated by *). The presence of Gd^{3+} causes significant peak broadening. (B) Size distribution profiles of p(DFO-AEA-*co*-OEGA-*co*-[Gd^{3+}]VDMD) star polymer (D_h = 13 nm), as determined by dynamic light scattering (DLS) (n=3). D_h: Number-average hydrodynamic diameter.

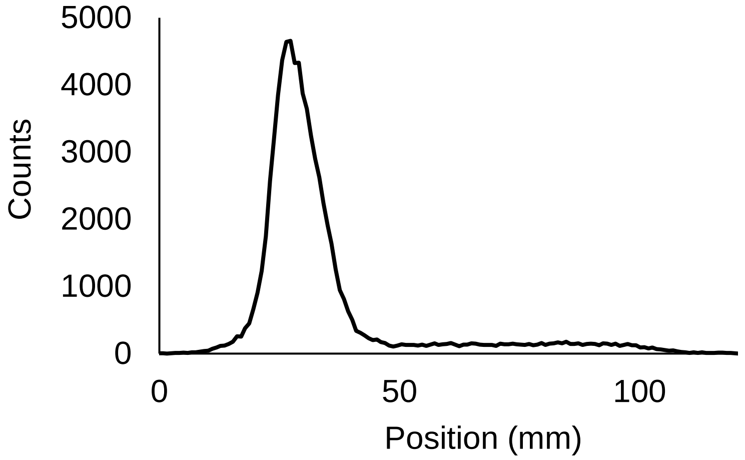

Figure S10. Radiochemical purity of star polymer **8** (p([^{89}Zr]Zr-DFO-AEA-*co*-OEGA-*co*-[Gd^{3+}]VDMD)) was >99% (DCY = >99%, MA = >290 GBq/µmol), as determined by instant thin layer chromatograpy (iTLC).

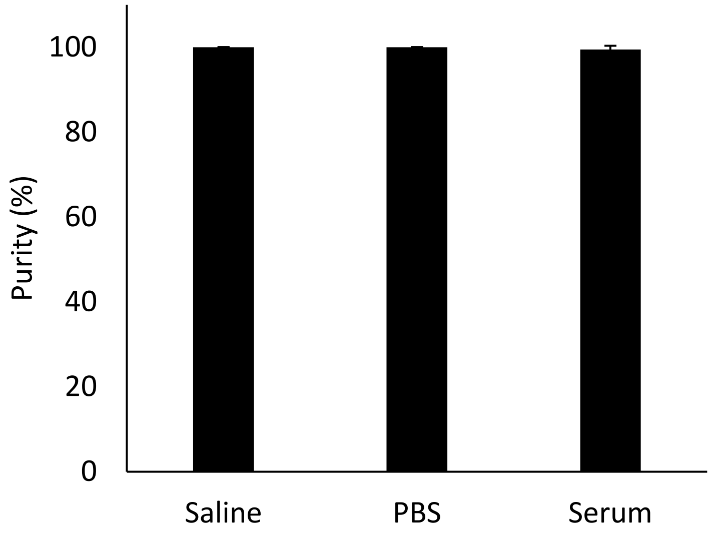

Figure S11. *In vitro* stability of star polymer **8** (p([^{89}Zr]Zr-DFO-AEA-*co*-OEGA-*co*-[Gd^{3+}]VDMD)) in saline, PBS and human serum (n = 3). Purity was measured by iTLC after overnight incubation at 37°C.

A

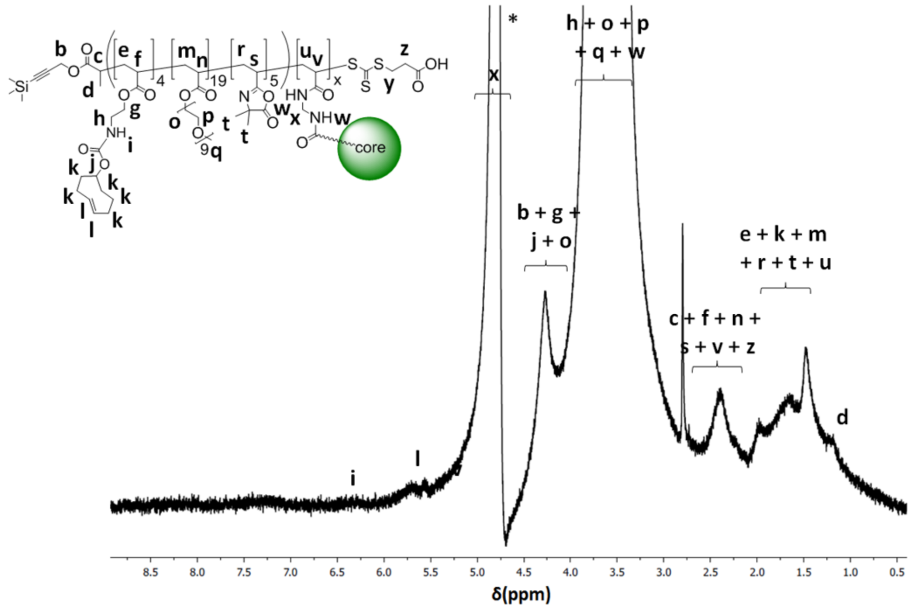

B

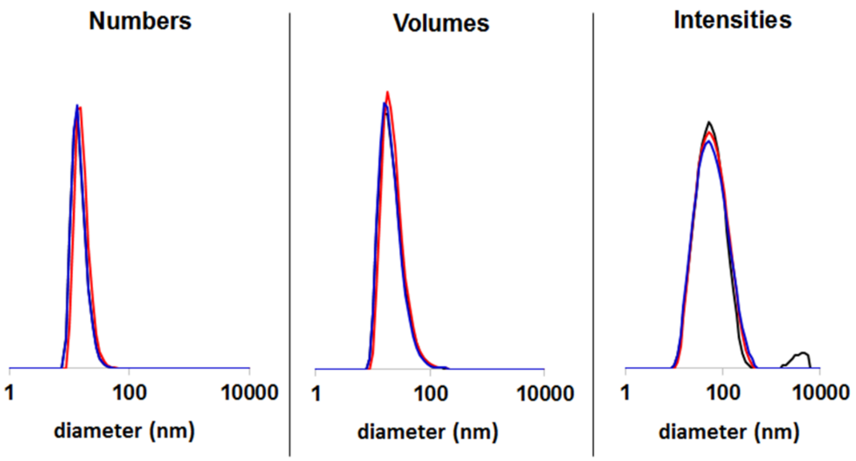

Figure S12. (A) ^{1}H NMR spectrum of p(TCO-AEA-*co*-OEGA-*co*-[Gd^{3+}]VDMD) star polymer (**9**) in D_2O (residual solvent peak indicated by *). The presence of Gd^{3+} causes significant peak broadening. (B) Size distribution profiles of p(TCO-AEA-*co*-OEGA-*co*-[Gd^{3+}]VDMD) star polymer (**9**; D_h = 12 nm), as determined by dynamic light scattering (DLS) (n=3). D_h: Number-average hydrodynamic diameter.

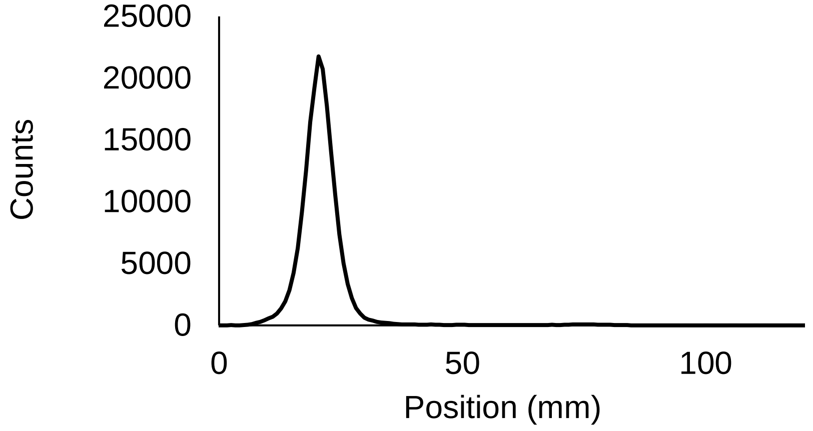

Figure S13. Radiochemical purity of star polymer **11** (p([^{177}Lu]Lu-DPAEA-*co*-OEGA-*co*-[Gd^{3+}]VDMD)) was >99% (DCY = >95%, MA = 615 GBq/µmol), as determined by instant thin layer chromatography (iTLC).

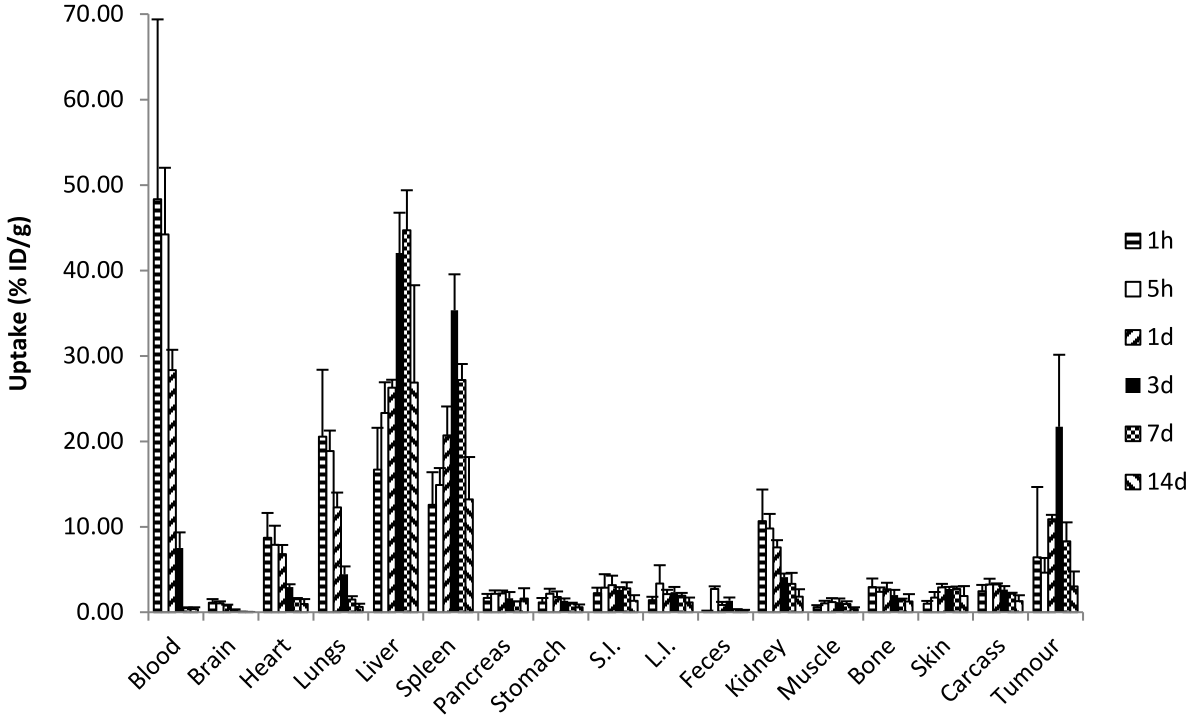

Figure S14. Full biodistribution profile of ^{177}Lu-labelled nanostars (~0.8 MBq, MA = ~5 GBq/µmol) at different timepoints after injection in BALB/c mice allografted with CT26 colon cancer cells. Highest tumour accumulation was observed after 3 days, as well as accumulation in liver and spleen. Seeming decrease in tumour accumulation after 3 days is likely an artefact of the rapidly growing tumour model without a sustained administration of radiopharmaceutical in combination with actual washout S.I.: small intestine, L.I.: large intestine.

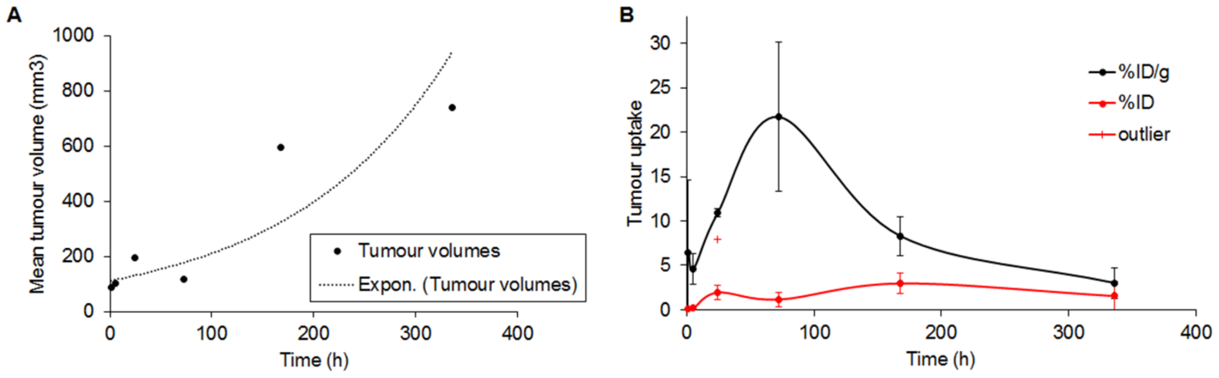

Figure S15. (A) Mean tumour growth in time after injection of the ^{177}Lu-labelled nanostars. (B) Accumulation of the ^{177}Lu-labelled nanostars in the tumour over time, calculated in %ID/g or %ID (+: outlier).

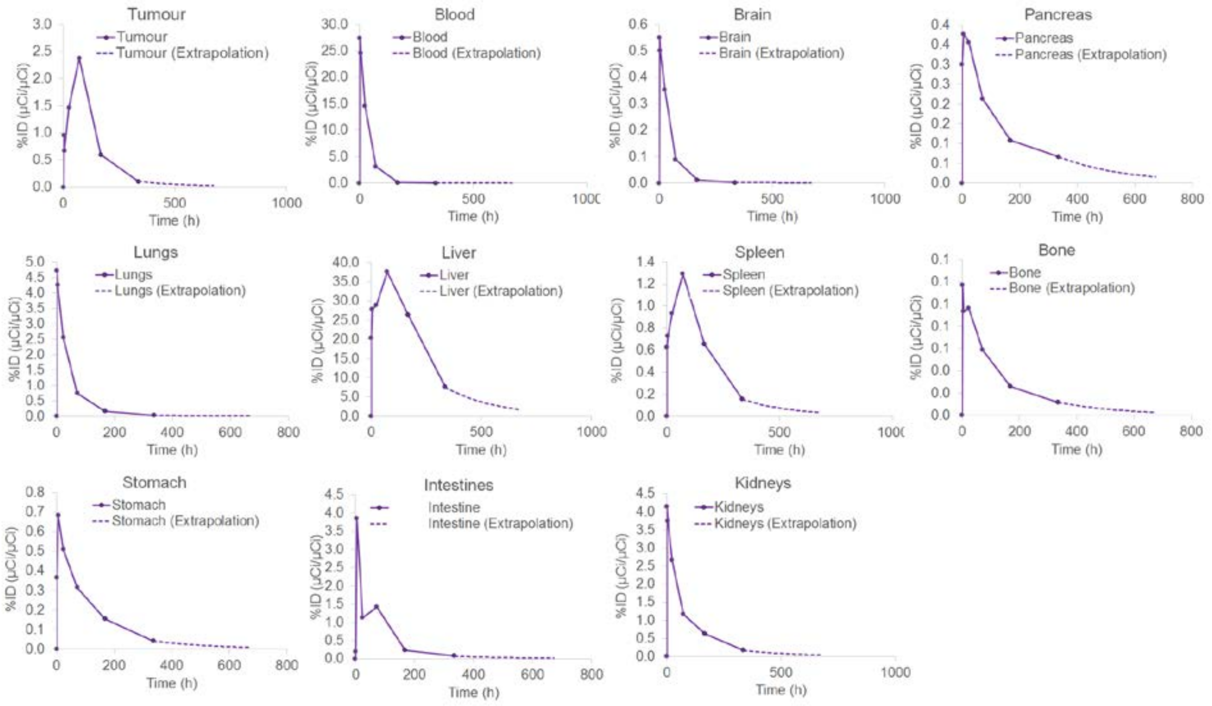

Figure S16. Murine time-activity curves per organ. Trapezoidal integration was used to calculate the time-integrated activity coefficient per organ. Clearance after the last time point (dotted line, ‘extrapolation') was estimated by assuming clearance was due to radioactive decay only.

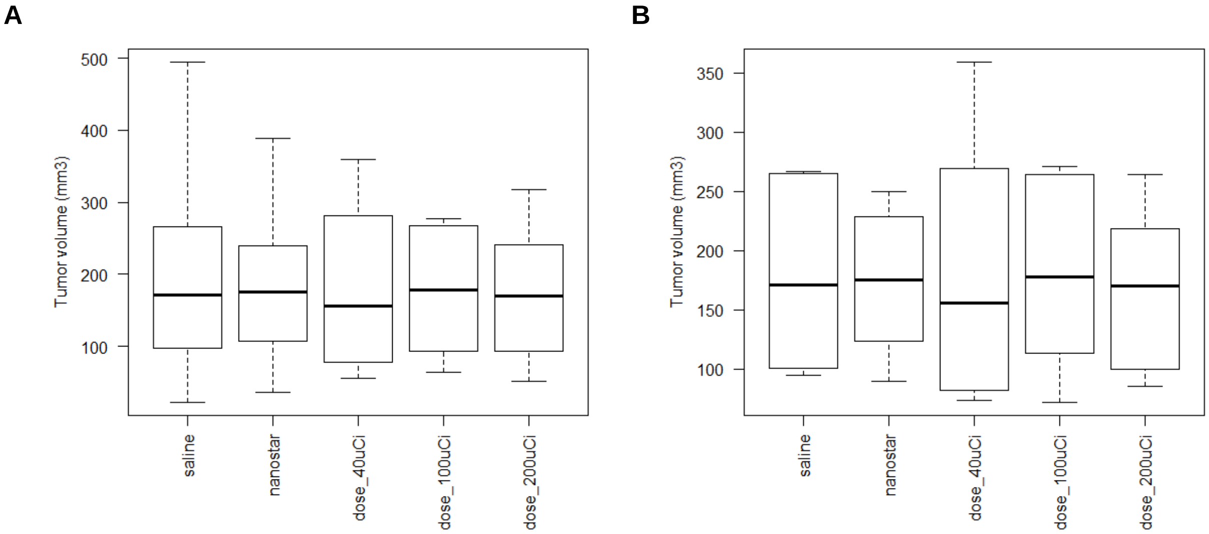

Figure S17. (A) Average tumour volumes of each therapy cohort after randomization (n = 8 per cohort). (B) Average tumour volumes of each therapy cohort after exclusion of the outliers – being the two mice with the smallest and largest tumour volumes.

Table S1. Dosimetry and absorbed dose estimations in mice, calculated from biodistribution data of the ^{177}Lu-labelled nanostars in BALB/c mice isografted with CT26 colon cancer cells.

**Organ**	**Total (Gy/MBq)**	**Estimated dose (Gy)**		
		**1.5 MBq**	**3.7 MBq**	**7.4 MBq**
Blood	1.63	2.41	6.03	12.06
Heart	0.54	0.80	2.00	4.00
Lungs	0.85	1.26	3.16	6.31
Liver	6.87	10.17	25.42	50.84
Spleen	4.55	6.74	16.84	33.68
Pancreas	0.32	0.47	1.18	2.35
Stomach	0.22	0.32	0.81	1.62
Small Intestine	0.14	0.21	0.52	1.03
Colon	0.14	0.21	0.52	1.03
Kidneys	0.78	1.16	2.90	5.79
Bone (All)	0.34	0.50	1.25	2.50
Tumor	1.94	2.87	7.17	14.33
Carcass	0.43	0.64	1.60	3.20
Brain	0.05	0.07	0.17	0.35

Table S2. Dosimetry and absorbed dose estimations for the 70 kg Standard Man model, calculated from biodistribution data of the ^{177}Lu-labelled nanostars in BALB/c mice isografted with CT26 colon cancer cells.

**Organ**	**Total (mGy/MBq)**	**Estimated dose (Gy)**	
		**5.0 GBq**	**7.4 GBq**
Adrenals	0.21	1.05	1.55
Brain	0.02	0.10	0.15
Esophagus	0.17	0.85	1.26
Eyes	0.14	0.70	1.04
Gallbladder Wall	0.23	1.15	1.70
Left colon	0.18	0.90	1.33
Small Intestine	0.18	0.90	1.33
Stomach Wall	0.21	1.05	1.55
Right colon	0.19	0.95	1.41
Rectum	0.15	0.75	1.11
Heart Wall	0.57	2.85	4.22
Kidneys	0.34	1.70	2.52
Liver	2.73	13.65	20.20
Lungs	0.36	1.80	2.66
Pancreas	0.16	0.80	1.18
Prostate	0.15	0.75	1.11
Salivary Glands	0.14	0.70	1.04
Red Marrow	0.21	1.05	1.55
Osteogenic Cells	0.55	2.75	4.07
Spleen	1.77	8.85	13.10
Testes	0.14	0.70	1.04
Thymus	0.15	0.75	1.11
Thyroid	0.15	0.75	1.11
Urinary Bladder Wall	0.15	0.75	1.11
Total Body	0.24	1.20	1.78
